# Ultrasensitive Room Temperature Infrared Photodetection Using a Narrow Bandgap Conjugated Polymer

**DOI:** 10.1002/advs.202304077

**Published:** 2023-10-27

**Authors:** Chih‐Ting Liu, Jarrett Vella, Naresh Eedugurala, Paramasivam Mahalingavelar, Tyler Bills, Bernardo Salcido‐Santacruz, Matthew Y. Sfeir, Jason D. Azoulay

**Affiliations:** ^1^ School of Chemistry and Biochemistry and School of Materials Science and Engineering Georgia Institute of Technology Atlanta GA 30332 USA; ^2^ Sensor Directorate Air Force Research Laboratory Wright‐Patterson Air Force Base Dayton OH 45433 USA; ^3^ Photonics Initiative Advanced Science Research Center City University of New York New York NY 10031 USA; ^4^ Department of Chemistry The Graduate Center City University of New York New York NY 10016 USA; ^5^ Department of Physics The Graduate Center City University of New York New York NY 10016 USA

**Keywords:** blackbody, diradicals, donor–acceptor conjugated polymers, high‐spin organic materials, infrared photodetectors, organic semiconductors

## Abstract

Photodetectors operating across the short‐, mid‐, and long‐wave infrared (SWIR–LWIR, *λ* = 1–14 µm) underpin modern science, technology, and society in profound ways. Narrow bandgap semiconductors that form the basis for these devices require complex manufacturing, high costs, cooling, and lack compatibility with silicon electronics, attributes that remain prohibitive for their widespread usage and the development of emerging technologies. Here, a photoconductive detector, fabricated using a solution‐processed narrow bandgap conjugated polymer is demonstrated that enables charge carrier generation in the infrared and ultrasensitive SWIR–LWIR photodetection at room temperature. Devices demonstrate an ultralow electronic noise that enables outstanding performance from a simple, monolithic device enabling a high detectivity (D*, the figure of merit for detector sensitivity) >2.44 × 10^9^ Jones (cm Hz^1/2^ W^−1^) using the ultralow flux of a blackbody that mirrors the background emission of objects. These attributes, ease of fabrication, low dark current characteristics, and highly sensitive operation overcome major limitations inherent within modern narrow–bandgap semiconductors, demonstrate practical utility, and suggest that uncooled detectivities superior to many inorganic devices can be achieved at high operating temperatures.

## Graphical abstract

A solution‐processed narrow bandgap conjugated polymer enables ultrasensitive infrared photodetection at room temperature. A simple, monolithic photoconductive device demonstrates a low electronic noise, enabling a detectivity of >10^9^ Jones using the ultralow flux of a blackbody that mirrors the background emission of objects. This performance suggests practical utility, and that uncooled detectivities superior to inorganic devices are achievable at high operating temperatures.

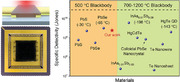

## 1 Introduction

Modern infrared (IR) photodetectors operating across the short‐wave IR (SWIR, 1–3 µm), mid‐wave IR (MWIR, 3–5 µm), and long‐wave IR (LWIR, 8–14 µm) sensitively distinguish between the low‐intensity blackbody emission of objects and background irradiance, forming the basis for modern detection and imaging technologies.^[^ ^1^, ^2^ ^]^ Emerging applications having global societal impacts in renewable energy, environmental monitoring, healthcare, information science, building and machine automation, and consumer applications require ubiquitous, uncooled, and low‐cost detectors that operate with the same inherent sensitivity.^[^ ^3^, ^4^ ^]^ However, today's blackbody‐sensitive detectors remain reliant on traditional narrow bandgap semiconductors. These include materials such as lead sulfide (PbS, *λ* = 1–3 µm) and lead selenide (PbSe, *λ* = 1–7 µm), II–VI and III–V compound semiconductors such as indium gallium arsenide (In_1‐x_Ga_x_As, *λ* = 1–2.4 µm) and indium antimonide (InSb, *λ* = 3–5 µm), and ternary alloys of mercury cadmium telluride (Hg_1‐x_Cd_x_Te, *λ* = 8–14 µm).^[^ ^2^ ^]^ Despite decades of development and the maturity and performance of semiconductors that form the basis for SWIR–LWIR electro‐optical/IR (EO/IR) systems, several drawbacks fundamentally limit their widespread usage and the development of critical emerging technologies. Most notably, these include expensive growth methods, complex manufacturing (e.g., multistep processes of physical or chemical evaporation, photolithography, complex die transfer, and bonding), the use of toxic materials (Hg, As, Pb), complex integration with silicon‐based electronics, and active cooling to reduce the thermal noise. The latter is particularly critical for narrow‐bandgap semiconductors operating in the MWIR–LWIR, where cryogenic cooling is required to achieve high performance.^[^ ^5^, ^6^ ^]^

To address these challenges, widespread efforts in scientific and engineering disciplines have explored the development and application of alternative narrow bandgap semiconductors. A primary goal has been the development of high‐operating temperature (HOT) photodetectors that enable direct integration with silicon‐based technologies, thereby benefitting from technological advancements in integrated circuit technologies. Leading semiconductor materials candidates that operate in the SWIR–LWIR are largely derived from low‐dimensional materials^[^ ^7^ ^]^ such as colloidal quantum dots (CQDs),^[^ ^8^, ^9^ ^]^ 2D materials,^[^ ^10^, ^11^, ^12^, ^13^ ^]^ and transition metal dichalcogenides (TMDCs).^[^ ^14^, ^15^, ^16^ ^]^ These alternative semiconductors demonstrate high carrier mobilities leading to considerable dark currents, which results in low signal‐to‐noise ratios and correspondingly low sensitivity. However, the creation of heterojunction photodetectors has offered opportunities to suppress dark currents and enable sensitive ambient temperature IR detection.^[^ ^17^, ^18^ ^]^ While emerging detectors with sensitivities comparable to those of commercial technologies have been reported, this performance is often considerably overestimated by using strong light sources such as IR lasers and high‐temperature blackbodies. The response of these detectors to blackbody radiation at low photon fluxes, which mirror the incident power levels emanating from real‐world objects is indicative of the sensitivity needed for practical application. Although these measurements represent long‐standing test methods used for the qualification of commercial technologies such as focal plane arrays (FPAs),^[^ ^19^ ^]^ they are often overlooked when developing new materials. However, ambient temperature blackbody sensitive SWIR–LWIR operation has been demonstrated using diverse materials such as mercury chalcogenide QDs and CQDs,^[^ ^20^ ^]^ black phosphorus (b‐P) and black arsenic phosphorus (b‐AsP),^[^ ^21^ ^]^ low‐dimensional Te,^[^ ^22^ ^]^ PbSe nanocrystals,^[^ ^23^ ^]^ and various combinations of materials such as heterojunctions of HgCdTe and b‐P,^[^ ^24^ ^]^ Te and graphene,^[^ ^25^ ^]^ MoS_2_/InGaAs,^[^ ^26^ ^]^ and graphene on epitaxial HgCdTe.^[^ ^27^ ^]^ However, challenges associated with low‐light absorption, challenging fabrication, and instability are major impediments toward the development of room‐temperature long‐wavelength IR optoelectronics based on low‐dimensional materials.

Conjugated polymers (CPs) offer simultaneous solutions to the aforementioned challenges owing to their ease of synthesis and tunability, very high absorption coefficients, seamless integration with silicon electronics, uncooled operation, and low‐cost, large‐area, and scalable fabrication.^[^ ^4^, ^28^ ^]^ Moreover, CPs are comprised of non‐toxic and Earth‐abundant materials, are tolerant toward structural defects and disorder, and offer the capability to be fabricated into diverse and mechanically compliant form factors. These attributes offer opportunities for emerging technologies that are unavailable from other semiconductors. Although fundamental limitations have largely restricted the long‐wavelength operation of CPs to the NIR–SWIR, recent reports have demonstrated donor–acceptor (DA) CPs with narrow bandgaps spanning the SWIR–LWIR, whose novel electronic structures enable optical to electrical transduction of IR light.^[^ ^29^ ^]^ Furthermore, structural and energetic heterogeneities and low carrier mobilities inherent within these materials impart unique carrier properties, offering extremely low noise spectral density at room temperature. Here, we demonstrate an ultrasensitive photoconductive detector that uses a DA CP active layer and offers broadband operation from the SWIR–LWIR. Devices demonstrate an ultralow dark noise of 179 fA Hz^−1/2^ when operating under an applied +5 V DC bias, which rivals or exceeds cooled commercial technologies and overcomes a major limitation inherent within modern narrow–bandgap semiconductors. This enables outstanding performance using a simple monolithic device architecture, enabling a high specific detectivity >2.44 × 10^9^ Jones using the ultralow flux of a 500 °C blackbody that mirrors the background emission of objects.

## 2 Results and Discussion

**Figure 1:**
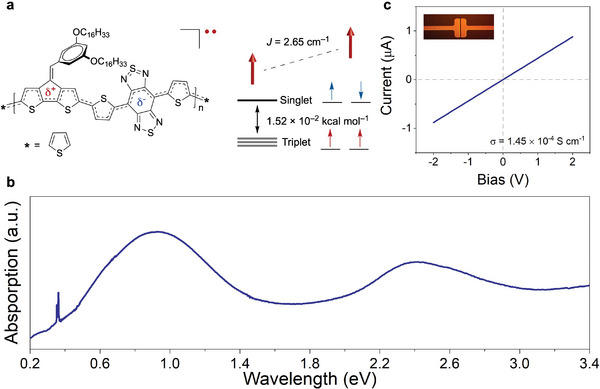
Structure and solid‐state properties of the IR absorbing conjugated polymer. a) Molecular and electronic structure of the narrow bandgap polymer. The measured magnetic properties show a high‐to‐low spin energy gap of 1.52 × 10^−2^ kcal mol^−1^ and exchange coupling constant (*J*) of 2.65 cm^−1^ that is consistent with a parallel spin alignment. b) Absorption spectra of a thin film spin cast from chlorobenzene onto an NaCl substrate. c) Current–voltage characteristics in a 60 µm × 1 mm channel with a conductivity (*σ*) of 1.45 × 10^−4^ S cm^−1^.

**Figure** **1**a displays the molecular and electronic structure of the copolymer used in this study, poly((4‐(3,5‐bis(hexadecyloxy)benzylidene)−4*H*‐cyclopenta[2,1‐*b*:3,4‐*b″*]dithiophene‐2,6‐diyl)‐*alt*‐4,7‐bis(5‐thiophen‐2‐yl)−2*λ* ^4^ *δ* ^2^‐benzo[1,2‐*c*;4,5‐*c′*]bis[1,2,5]thiadiazole). The polymer was synthesized using a modified microwave‐assisted Stille cross‐coupling copolymerization that enabled in situ chain‐end functionalization with thiophenes, which was essential for improving the solubility and processability of the material.^[^ ^30^, ^31^ ^]^ Full details regarding the synthesis and characterization can be found in the Supporting Information. The polymer incorporates a cross‐conjugated 4*H*‐cyclopenta[2,1‐*b*:3,4‐*b*″]dithiophene (CPDT) donor capable of raising the highest occupied molecular orbital (HOMO), paired with a strong thiophene‐flanked benzo[1,2‐*c*;4,5‐*c’*]bis[1,2,5]thiadiazole (BBT) acceptor capable of lowering the lowest unoccupied molecular orbital (LUMO). Ancillary phenyl units on the donor enable strategic positioning of solubilizing −OC_16_H_33_ substituents to minimize backbone torsion and promote a high degree of electronic coherence.

This polymer exhibits a broad infrared absorption extending from the SWIR−LWIR that enables its use in long‐wavelength photodetectors. We measure the absorption bandwidth of our polymer using a thin film fabricated by spin coating from a 10 mg mL^−1^ chlorobenzene solution onto an NaCl substrate (Figure 1b). The absorption maximum (*λ*_max_) of 1.35 µm in the SWIR is related to the extensive *π*‐conjugation in the material. However, an additional manifold of low‐energy electronic states is apparent from a broad absorption profile that extends throughout the MWIR–LWIR. These measurements give an upper bound for the optical bandgap (*E*_g_^opt^) of 0.10 eV (Figure 1b). The narrow bandgap is characteristic of DA CPs with open‐shell electronic structures in which weakly paired or unpaired electrons evolve from strong configuration mixing between the frontier molecular orbitals (i.e., HOMO and LUMO mixing).^[^ ^32^, ^33^, ^34^ ^]^ As shown in Figure 1a, this results in an orbital manifold in which valence electrons occupy singly occupied molecular orbitals (SOMOs), which contrasts with closed‐shell CPs that accommodate their *π*‐electrons in bonding orbitals. Thus, low‐energy optical transitions emerge involving unpaired electrons that occupy nearly degenerate SOMOs. In open‐shell DA CPs, significant internal charge transfer character between electron‐rich (donor) and electron‐deficient (acceptor) heterocyclic units and extended *π*‐conjugation result in extensive spin polarization throughout the *π*‐conjugated backbone. This topological structure determines the degree of spin pairing, physicochemical properties, and (opto)electronic functionality.^[^ ^32^, ^33^, ^34^, ^35^, ^36^, ^37^ ^]^ Superconducting interference device (SQUID) magnetometry and electron spin resonance (ESR) spectroscopy are consistent with the polymer adopting a high‐spin ground state with a nearly degenerate singlet–triplet energy splitting (Δ*E*_ST_) of 1.52 × 10^−2^ kcal mol^−1^ and exchange coupling coefficient (*J*) of 2.65 cm^−1^ between weakly interacting and ferromagnetically aligned spins (see Figure 1a; Figure S4, Supporting Information for full details).^[^ ^38^ ^]^ Density functional theory (DFT) calculations confirm our assignment that optical transitions throughout the IR (Figure S14, Supporting Information) are associated with the evolution of an open‐shell structure as a function of chain length (Figures S7–S13, Table S2, and Section S3.9, see Supporting Information for full details).

To evaluate the transport properties, thin‐polymer films were spin‐coated onto *n*‐octadecyltrichlorosilane (OTS)‐treated SiO_2_/Si substrates (electrodes: Cr ≈5 nm, Au ≈60 nm, length (*L*) = 60 µm and channel width (*W*) = 1 mm) to construct bottom‐gate, bottom contact (BGBC) devices (Figure 1c, inset). The device shows linear *I‒V* characteristics consistent with Ohmic transport and a room‐temperature conductivity (*σ*_RT_) of 1.45 × 10^−4^ S cm^−1^ (Figure 1c). The device demonstrated *p*‐type field‐effect transistor (FET) behavior with a hole mobility (*µ*) of 1.68 × 10^−4^ cm^2^ V^−1^ s^−1^. The transfer curve (Figure S5b, Supporting Information) does not demonstrate an off state, indicating the presence of free carriers. In closed‐shell conjugated polymers, extrinsic doping processes are required to achieve IR absorption and improve the conductivity to levels suitable for applications, giving rise to properties that are problematic for many optoelectronic technologies. These include high chemical reactivity, material and device instability, processing and performance limitations, and incompatibility with substrates and electronic components. The broad, long‐wavelength IR absorption and intrinsic electrical conductivity are achieved in the absence of doping and result in charge–neutral polymeric species that overcome these inherent limitations.

**Figure 2:**
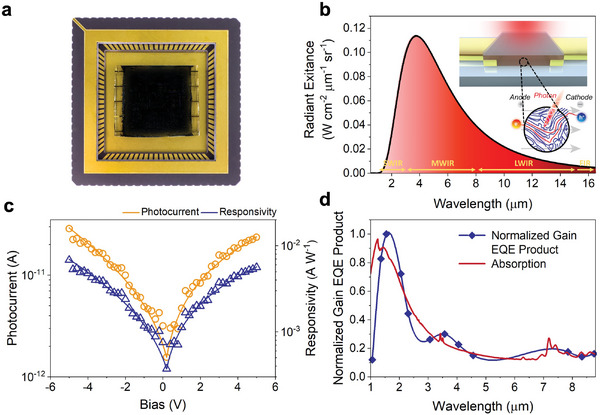
Device structure and photocurrent response. a) Photoconductive devices mounted in a ceramic leadless chip carrier. b) Calculated exitance of a 500 °C blackbody radiator with a schematic illustration of the 60 µm × 1 mm detector active area and the mechanism of operation (inset). c) Photocurrent generated under irradiation with a 500 °C blackbody and responsivity as a function of applied bias. d) Spectral response of the photoconductor measured using a series of narrow bandpass filters and absorption profile of the polymer (red line). The narrow bandpass filters are represented by the blue dots, while the blue line is a guide to the eye.

The distinct chemical, optical, and electronic features of the open‐shell polymer—strong IR absorption, intrinsic electrical conductivity, solution processability, and high chemical stability enabled the fabrication of a photoconductive detector. In this device architecture, absorption of IR photons by the detector material generates transient charge carriers above an equilibrium concentration (**Figure** **2**a). Under the action of an external bias, this temporarily lowers the resistance of the detector. The lowered resistance is measured as a temporary increase in current or drop in voltage. Thus, the photoconductor in Figure 2 consists of pre‐patterned gold electrodes on quartz substrates that are separated by a 60 µm × 1 mm spacing, which acts as the detector active area. Transmission lines leading away from each electrode act as contacts for electrical measurements (Figure 2b). A thick polymer film (≈1.1 µm) was drop‐cast on the electrodes followed by encapsulation with a 100 nm amorphous alumina layer (Figure 2b, inset) that is largely transparent throughout the SWIR and MWIR, with only slight LWIR attenuation. The substrate was then epoxied into a ceramic leadless chip carrier (LCC) and after curing, ultrasonically extruded gold wire bonds were attached to the contact pads and to appropriate traces on the LCC. The LCC was then placed into a custom fabricated printed circuit board for electrical characterization (see Supporting Information).

Current–voltage (*I–V*) measurement in the dark of the device in Figure 2a yields a straight line that is symmetric at 0 V (Figure S5a, Supporting Information). At a bias level of +5 V DC, the dark current is 2.23 µA, giving a resistance (bias voltage/dark current) of 2.24 MΩ. To gauge the sensitivity of the detector, it was illuminated with a 500 °C cavity blackbody radiator chopped at 200 Hz. The calculated radiant exitance (Figure 2b) features a maximum intensity at *λ* = 3.74 µm. The signal was read out using a current pre‐amplifier connected to a lock‐in amplifier. Using this setup, the polymer generated a photocurrent symmetric at ≈0 V, with a +5 V DC value of 41.1 pA (Figure 2c). This corresponds to a responsivity of ℛ = 17.8 mA W^−1^ (Figure 2c). Repeated measurements of the detector showed the production of photocurrent after more than one year (Figure S6, Supporting Information).

To determine precisely what wavelengths contribute to the responsivity, the product of gain and external quantum efficiency (*ηG*) was measured by placing a series of narrow bandpass filters between the blackbody radiator and detector with the results presented in Figure 2d. Significantly, the absorption spectrum closely mirrors the *ηG* curve over the measured range, with its maximum response near *λ*_max_ at ≈ 1.5 µm. These data allow us to rule out both a photodiode mechanism, which would exhibit a nonlinear dark *I*–*V* curve, and a thermal detector such as a microbolometer, which would exhibit a spectrally flat photoresponse. Beyond 3 µm, the photocurrent is affected by readout noise of the *ηG* detection system, producing artificial local maxima that are not present for samples with a higher efficiency measured using the same setup.^[^ ^39^ ^]^

**Figure 3:**
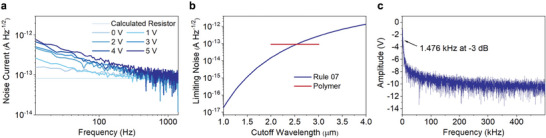
Noise spectral measurements. a) Noise spectral density for the photoconductive detector. Amplifier noise has been subtracted in quadrature curves. b) Comparison of polymer noise levels to the state‐of‐the‐art using Rule 07. c) Bode plot of the photoconductive detector showing a 3 dB bandwidth of 1.476 kHz.

The noise equivalent power (NEP) defines the minimum optical power that a detector can distinguish from noise and is a key metric that quantifies the performance and sensitivity of a photodetector. Because a photodetectors performance varies with its area (*A*_d_) and the noise equivalent bandwidth (Δ*f*), specific detectivity (D*) is used to compare performance between different detector technologies, with D* = (*A*_d_Δ*f*)^1/2^/NEP, given in units of Jones (cm Hz^1/2^ W^−1^). To calculate D*, we first measured the noise spectral density (*N*_0_, **Figure** **3**a). In contrast to a typical dark *I*–*V* curve where current is measured as a function of variable DC bias, noise spectral density is measured by holding a DC bias level constant and dividing the resulting noise power by the measurement bandwidth. The noise spectral density curve is affected by ohmic barriers between electrodes and the polymer, by rates of carrier generation and recombination, and by thermally generated charge carriers, not simply by resistance as in a DC dark‐current measurement. Using the dark *I*–*V* curve (Figure S5a, Supporting Information), we previously calculated the detector resistance to be 2.24 MΩ. The expected Johnson noise (*i*_J_) of such a resistor, normalized for bandwidth, is (4*k*_B_ *T/R*)^1/2^, where *k*_B_ is Boltzmann's constant, *T* is detector temperature, and *R* is resistance, which gives the Johnson noise current of 86 fA Hz^−1/2^ for the detector. Indeed, *N*_0_ for all of the bias levels eventually reaches this fundamentally limiting noise level as the modulation frequency approaches 1000 Hz. At lower frequencies, the noise spectral density increases with bias level, suggesting it is limited by 1/*f* noise. A transition knee was not observed, preventing a definitive assignment. Regardless, at the blackbody chopping frequency of 200 Hz, *N*_0_ increases from 93.8 fA Hz^−1/2^ at +1 V DC to 179 fA Hz^−1/2^ at +5 V DC. Dividing the noise spectral density by the +5 V DC responsivity gives the noise equivalent power (NEP) of 10.1 pW Hz^−1/2^.

With this information, we can compare the photoconductor to state‐of‐the‐art noise levels for room temperature detectors. Rule 07 is an empirical guide that calculates state‐of‐the‐art noise current densities for photodetectors as a function of cutoff wavelength (*λ*_c_) for a given temperature.^[^ ^40^ ^]^ Rule 07 is based on shot noise‐limited HgCdTe (*λ*_c_ varies up to 18 µm) photodiodes. Photodiodes are limited by shot noise, while photoconductors are limited by Johnson noise. To make an even comparison, the Rule 07 dark current density was converted to shot noise (units of A Hz^−1/2^) through the relation (2*qJA*)^1/2^, where *q* is the fundamental charge, *J* is the dark current density calculated from Rule 07, and *A* is detector area. This way, the fundamentally limiting noise level for a photodiode can be made to a photoconductor (Figure 3b). Depending on the cutoff wavelength of the photoconductor, its noise is approximately equal to or slightly better than a state‐of‐the‐art room temperature detector. Finally, we can use the noise spectral density measurements to calculate D*. This is typically calculated by D* = *(A*Δ*f)* ^1/2^ */*NEP, where Δ*f* is the noise equivalent bandwidth, and the NEP is given in W and has units of Jones (cm Hz^1/2^ W^−1^). While the function of Δ*f* is to normalize for bandwidth, the noise spectral density (*N*_0_) is already normalized and an alternate form can be used where D* = *ℛA* ^1/2^ */N*_0_, which gives D* = 2.44 × 10^9^ Jones.

The response time and 3 dB bandwidth are important figures of merit for photodetectors that are normally calculated using resistance–capacitance time constant measurements. However, an *I–V* phase angle of 0° indicates that the material is resistive and prevents such measurements. Instead, the rise time (*τ*_r_) was measured from the impulse response using a femtosecond (fs) laser operating at *λ* = 1.55 µm. The 150 fs laser pulse width ensures that only electronic processes are excited in the material and is significantly faster than most detectors can respond. It follows that the time the detector takes to go from 10% to 90% of its maximum photocurrent enables an accurate measurement of *τ*_r_, which is found to be 226 µs. This can be related to the 3 dB bandwidth of 1.55 kHz using *f* = (2*πτ*_r_)^−1^. Conversion of the optical impulse response to frequency space using a Fast Fourier Transform provides the photocurrent frequency spectrum (Figure 3c), which gives a 3 dB bandwidth of 1.476 kHz. This agrees closely with the optically measured *τ*_r_ obtained from the rise time.

**Figure 4:**
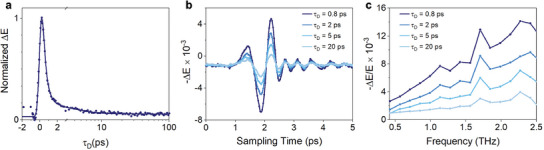
Time‐resolved THz spectroscopy of the high‐spin conjugated polymer thin film. a) The charge carrier dynamics are determined by measuring the differential transmission of a THz probe pulse after photoexcitation at 1400 nm. The kinetics are measured at the maximum of the waveform as the delay time (*τ*_D_) from the pump pulse is scanned. We observe an ultrafast decay with two primary components: *τ*_1_ < 0.5 ps (resolution limited by the instrument response function) and *τ*_2_ = 3.3 ps. b) The full waveform of the differential electric field normalized to the maximum of the ground state THz transmission is recorded at several characteristic values of *τ*_D_. c) The corresponding differential photoconductivity spectra for different delay times (*τ*_D_) shows that the photoconductivity is positive and increases with increasing frequency.

The photoconductive origin of our photodetectors is further confirmed using time‐resolved terahertz (THz) spectroscopy that serves as a contactless probe of the transient photoconductivity in the time‐domain.^[^ ^41^ ^]^ We find that photoexcitation of the high‐spin conjugated polymer thin film in the infrared leads to the rapid generation of charge carriers that transiently enhance the conductivity, consistent with device performance. In these measurements, a THz pulse is generated using optical rectification of ultrafast optical pulses, and the waveform is detected in the time domain using standard electro‐optic sampling techniques (See Section S3.4, Supporting Information).^[^ ^42^ ^]^ The photoconductivity is determined by modulating a short (≈100 fs) infrared pump pulse and measuring the differential THz transmission through a thin polymer film on a quartz substrate. Upon photoexcitation at 1.4 µm, we observe a rapid decrease in the transmitted THz amplitude (**Figure** **4**a), corresponding to an increasing photoconductivity signal. The charge carrier dynamics are determined by sampling the peak of the THz waveform and scanning the pump‐probe delay time (*τ*_D_). Here, we find that the rise of the charge carriers signal is limited by the instrument response function of our probe (<0.5 ps). Furthermore, we observe two primary decay components with time constants (Figure 4a). Approximately 90% of the photoconductivity signal decays within the instrument response function and a second weaker component has a time constant of ≈3.5 ps. The frequency dependence of the photoconductivity is determined by recording the full THz waveform at several characteristic values of *τ*_D_ (Figure 4b) and performing a Fourier transform (Figure 4c). Here, we find that the transient photoconductivity (−Δ*E*/*E*) increases as frequency increases over the measured range. A similar response has been observed in other disordered and polymer materials.^[^ ^43^, ^44^ ^]^

**Figure 5:**
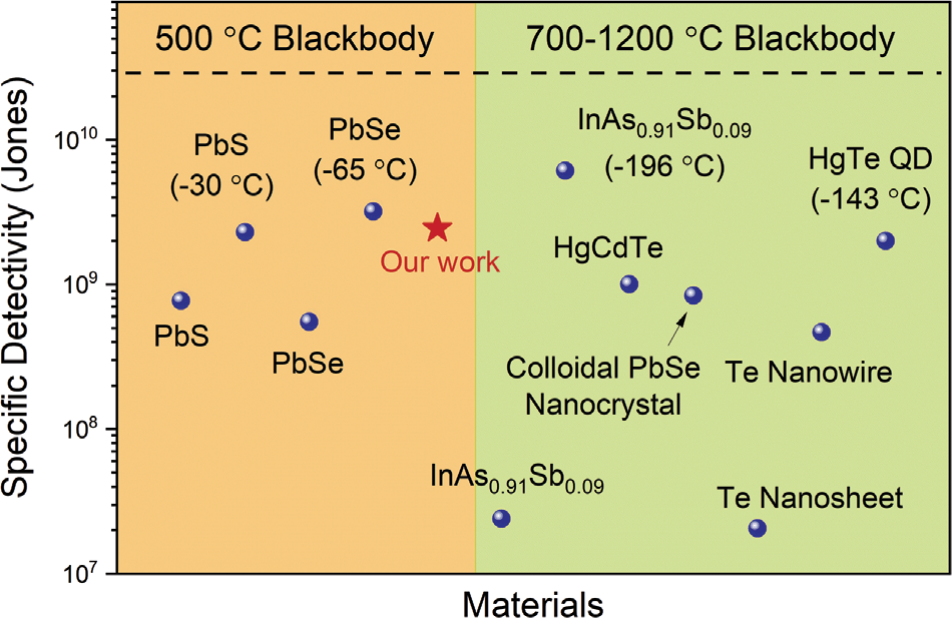
Comparison of the specific detectivity (D*) of our polymer detector (red star) with commercial photoconductive devices and those based on emerging semiconductor materials (blue dots). The materials on the left are tested using a 500 °C blackbody. Materials on the right were tested using blackbodies in the 700–1200 °C range. Operation temperature is room temperature or ambient conditions unless otherwise noted.

In the absence of optimization, this photoconductive detector is competitive with commercial blackbody‐sensitive technologies and emerging semiconductor materials (**Figure** **5**). For example, commercial uncooled PbS (*λ*  = 1–3 µm) and PbSe (*λ*  = 1.5–4.8 µm) show D* ≈10^8^ Jones when illuminated with a 500 °C blackbody and D* ≈10^9^ Jones when cooled to −30 and −65 °C, respectively.^[^ ^45^, ^46^ ^]^ The polymer shows a higher D* at 500 °C than other photoconductors measured using higher temperature blackbodies in the 700–1200 °C range. For example, commercial HgCdTe photoconductors (*λ*  = 2.2–10.6 µm) operating at room temperature show D* = 1 × 10^9^ Jones using an 800 °C blackbody.^[^ ^47^ ^]^ State‐of‐the‐art InAs_0.91_Sb_0.09_ photoconductors (*λ*  = 1–4 µm) grown using molecular beam epitaxy (MBE) show a blackbody (i.e., 727 °C) D* of 2.4 × 10^7^ and 6.1 × 10^9^ Jones at room temperature and −196 °C, respectively.^[^ ^48^ ^]^ Photoconductors fabricated from low‐dimensional Te in the form of nanosheets (*λ*  = 0.5–2.5 µm) and nanowires (*λ*  = 0.5–2.5 µm) show room temperature D* ≈2 × 10^7^ and ≈4 × 10^8^ Jones, when illuminated with a 927 °C blackbody.^[^ ^22^ ^]^ Solution processable PbSe nanocrystals show D* = 8.36 × 10^8^ Jones when illuminated with a 900 °C blackbody at room temperature.^[^ ^23^ ^]^ The polymer also performs better than QDs based on mercury chalcogenides. For example, HgTe (*λ*  = 3–5 µm) shows D* ≈2 × 10^9^ Jones at room temperature using a 1200 °C blackbody.^[^ ^49^ ^]^

## 3 Conclusion

This work demonstrates a photoconductive detector, fabricated using a solution‐processed narrow bandgap conjugated polymer that enables charge carrier generation in the infrared and ultrasensitive SWIR–LWIR photodetection at room temperature. The very narrow bandgap and open‐shell electronic structure impart intrinsic electrical conductivity and low noise characteristics. Combined with the robust chemical stability, straightforward synthesis, and solution processability of the polymer, these attributes coalesce to enable outstanding performance from a simple, monolithic device enabling a high D* > 10^9^ Jones using the ultralow flux of a 500 °C blackbody that mirrors the emission of objects. This performance exceeds commercial photoconductive technologies and the performance of emerging semiconductor materials. We demonstrate for the first time that open‐shell conjugated polymers offer a rare combination of advantageous properties such as broad spectral coverage, ease of fabrication, ultra‐low dark currents, and highly sensitive operation that overcomes major limitations inherent within modern narrow–bandgap semiconductors applied in photodetectors. Furthermore, these results demonstrate the practical utility and suggest that uncooled D* superior to many inorganic devices can be achieved at high operating temperatures.

## Conflict of Interest

The authors declare no conflict of interest.

## Supporting information

Supporting Information

## Data Availability

The data that support the findings of this study are available in the supplementary material of this article.
