# Supplementary material for: Ultrasensitive Room Temperature Infrared Photodetection Using a Narrow Bandgap Conjugated Polymer
Source: Adv Sci (Weinh). 2023 Oct 27;10(36):2304077. doi: 10.1002/advs.202304077 (PMC10754133; doi:10.1002/advs.202304077)
Supplement: Supplementary file 1 — Supporting Information [file ADVS-10-2304077-s001.pdf]

ADVANCED SCIENCE
Open Access

Supporting Information

for *Adv. Sci.*, DOI 10.1002/advs.202304077

Ultrasensitive Room Temperature Infrared Photodetection Using a Narrow Bandgap Conjugated Polymer

*Chih-Ting Liu, Jarrett Vella, Naresh Eedugurala, Paramasivam Mahalingavelar, Tyler Bills, Bernardo Salcido-Santacruz, Matthew Y. Sfeir and Jason D. Azoulay**

Supplemental Information for

Ultrasensitive Room Temperature Infrared Photodetection Using a Narrow Bandgap Conjugated Polymer

Chih-Ting Liu,[†] Jarrett Vella,[†] Naresh Eedugurala, Paramasivam Mahalingavelar, Tyler Bills, Bernardo Salcido-Santacruz, Matthew Y. Sfeir, and Jason D. Azoulay[*]

1. General Remarks
2. Synthesis
3. Experimental section
3.1. UV-Vis-NIR and Fourier Transform Infrared Spectroscopy
3.2. Electrochemistry
3.3. Conductivity and Transport Measurements
3.4. Terahertz Measurements
3.5. Detector Measurements
3.6. Grazing-Incidence wide-angle x-ray scattering (GIWAXS)
3.7. Electron Paramagnetic Resonance Spectroscopy
3.8. Superconducting Quantum Interference Device (SQUID) Magnetometry
3.9. Computational Methods
4. Supplemental figures S1–S16
5. Supplemental references

1. General Remarks

All manipulations of air/and or moisture sensitive compounds were performed under an inert atmosphere using standard glove box and Schlenk techniques. Reagents, unless otherwise specified, were purchased from Fisher Scientific and used without further purification. Solvents were degassed and dried over 4 Å molecular sieves prior to use. 1,1,2,2-Tetrachloroethane-d_2 and chloroform-*d* were purchased from Cambridge Isotope Laboratories and used as received. Tetrakis(triphenylphosphine)palladium(0) was purchased from Strem Chemicals and used as received. 4,7-bis(5-bromothiophen-2-yl)-2$\lambda^4\delta^2$ -benzo[1,2-*c*;4,5-*c′*]bis[1,2,5]thiadiazole[1] and (4-(3,5-bis(hexadecyloxy)benzylidene)-4*H*-cyclopenta[2,1-*b*:3,4-*b′*]dithiophene-2,6-diyl)bis(trimethylstannane)[2] were prepared according to literature procedures. ^{1}H and ^{13}C NMR spectra were collected on a Bruker Avance III 600 MHz spectrometer and chemical shifts, δ (ppm) were referenced to the residual solvent impurity peak of the solvent. Data are reported as: s = singlet, d = doublet, t = triplet, m = multiplet, br = broad, and coupling constant, (*J*) are reported in Hz. Flash chromatography was performed on a Teledyne Isco CombiFlash Purification System using Redi*Sep* Rf prepacked columns. Microwave assisted reactions were performed in a CEM Discover 2.0 microwave reactor. The number average molecular weight (M_n) and dispersity (*Đ*) were determined by gel permeation chromatography (GPC) using a universal calibration method at 160 °C in 1,2,4-trichlorobenzene (stabilized with 125 ppm of BHT) in an Agilent PL-GPC 220 high temperature GPC/SEC system using a set of three PLgel 13 µm Olexis columns. Polymer samples were dissolved at a concentration of 1–2 mg mL^{-1} in 1,2,4-trichlorobenzene with stirring for 4 hours at 160 °C.

2. Synthesis

Poly((4-(3,5-bis(hexadecyloxy)benzylidene)-4*H*-cyclopenta[2,1-*b*:3,4-*b′*]dithiophene-2,6-diyl)-*alt*-4,7-Bis(5-thiophen-2-yl)-2$\lambda^4\delta^2$ -benzo[1,2-*c*;4,5-*c′*]bis[1,2,5]thiadiazole): A microwave tube was loaded with (4-(3,5-bis(hexadecyloxy)benzylidene)-4*H*-cyclopenta[2,1-*b*:3,4-*b′*]dithiophene-2,6-diyl)bis(trimethylstannane) (0.115 g, 0.107 mmol, 1.05 equivalents) and 4,7-bis(5-bromothiophen-2-yl)-2$\lambda^4\delta^2$ -benzo[1,2-*c*;4,5-*c′*]bis[1,2,5]thiadiazole (0.053 g, 0.102 mmol, 1.0 equivalent). The tube was brought inside a nitrogen-filled glovebox, and 250 µL of a Pd(PPh3)4/xylenes stock solution (3.5 mol %) was added. The tube was sealed and subjected to the following reaction conditions in a microwave reactor with stirring: 120 °C for 5 min, 140 °C for 5 min, and 170 °C for 5 min. After polymerization, 1 mL xylenes and 0.25 equivalents (relative to the acceptor) of 2-(tributylstannyl)thiophene were added to the same tube inside a nitrogen-filled glovebox and stirred at 100 °C for 12 hours. Subsequently, 10 equivalents of 2-bromothiophene were added to the tube and stirred at 100 °C for another 12 hours. After this time, the reaction was allowed to cool, leaving a solid gelled material. The mixture was precipitated into methanol,

washed with acetone, and collected via filtration. The residual solid was loaded into an extraction thimble and washed successively (under a nitrogen atmosphere and in the absence of light) with methanol (2 hours), acetone (2 hours), hexanes (12 hours), a 1:1 mixture of hexanes and tetrahydrofuran (12 hours), and then acetone (2 hours). The polymer was dried *in vacuo* to give a black solid (71 mg, 63%). Data are as follows: M_n= 19.1 kg mol^{-1} and $Đ$ = 2.69; ^{1}H NMR (600 MHz, 1,1,2,2-tetrachloroethane-d_2, 413 K): δ 9.49–6.45 (br, 9H), 4.48–3.50 (4H, OCH_2), 2.45–1.12 (br, 57H), 1.12–0.76 (br, 6H); absorption: λ_{max} (solution, chlorobenzene, 25°C) = 1.32 μm, λ_{max} (thin film) = 1.35 μm,

3. Experimental Procedures

3.1. UV–vis–NIR and Fourier transform infrared spectroscopy

UV–Vis–NIR and Fourier transform infrared (FTIR) spectra were recorded from 0.375 to 3.30 μm and from 3.30 to 16.40 μm using a Cary 5000 UV–Vis–NIR spectrophotometer and Bruker VERTEX 80 FTIR spectrometer, respectively. Thin films were prepared by spin coating a chlorobenzene solution (10 mg mL^{-1}) onto quartz or NaCl substrates at 1,000 rpm.

3.2. Electrochemistry

Electrochemical characteristics were determined by cyclic voltammetry (50 mV s^{-1}) carried out on drop-cast polymer films at room temperature in degassed anhydrous acetonitrile with tetrabutylammonium hexafluorophosphate (0.1 M) as the supporting electrolyte. The working electrode was a platinum wire, the counter electrode was a platinum wire, and the reference electrode was Ag/AgCl. After each measurement the reference electrode was calibrated with ferrocene and the potential axis was corrected to the normal hydrogen electrode (NHE) using −4.75 eV for NHE.[3] The onset of oxidation was estimated at −4.95 eV and the onset of reduction was estimated at −4.10 eV.

3.3. Conductivity and Charge Transport Measurements

The conductivity and organic field-effect transistor (OFET) characteristics were evaluated using a typical bottom-gate, bottom-contact geometry. Silicon substrates were cleaned sequentially using 2% Hellmanex detergent in DI water, DI water, acetone, and isopropyl alcohol for 10 min each using sonication and then dried in an oven. The substrates were then treated in a UV/ozone cleaner for 20 min. The heavily n-doped silicon substrates with a 300 nm thermally grown SiO_2 dielectric were prepared as the bottom-gate electrode. The SiO_2 dielectric was passivated with octadecyl trichlorosilane ($CH_3(CH_2)_{17}SiCl_3$). 5 nm of chromium was thermally evaporated as an adhesive layer followed by 60 nm of gold at 10^{-6} torr using a shadow mask. The defined channel width was 1 mm, and the channel lengths were 30, 40, 50, 60, and 80 μm. A 10 mg mL^{-1} polymer solution was spin cast onto the substrate with pre-patterned Au electrodes.

Devices were tested on a probe station (Signatone 1160 series) inside a nitrogen-filled glovebox, and the data were recorded on a Keithley 4200 semiconductor characterization system. The conductivity measurements were conducted using a two-point probe method (source and drain, without gate) by sweeping the voltage from –2 to 2 V. The conductivity was determined from the equation (S1):

$$\sigma = \frac{I}{V} \times \frac{L}{WT} \qquad \text{(Eq. S1)}$$

where σ, I, V, L, W, and T represent conductivity, current, voltage, channel length, channel width, and polymer layer thickness (measured by AFM), respectively.[4] The hole mobility was extracted from the linear region of the transfer curve in a transistor geometry based on the equation (S2):

$$\mu = \frac{L}{WC_iV_D} \frac{\Delta I_D}{\Delta V_G} \qquad \text{(Eq. S2)}$$

where μ, L, W, C_i, V_D, I_D, *and* V_G represent the mobility, channel length, channel width, capacitance of the dielectric layer (300 nm thick SiO_2 layer), drain voltage, drain current, and gate voltage, respectively.[4]

3.4. Terahertz Measurements

Terahertz measurements were performed using the standard time-domain methods employing optical rectification and electro-optic sampling. Briefly, we generate the THz probe pulse using a ZnTe crystal pumped by the fundamental output of an amplified Ti:Sapphire laser (35 fs pulse width; 2kHz repetition rate). The resulting THz pulse is passed through a drop cast polymer film on a crystalline quartz substrate and focused into an electro-optical crystal. The THz bandwidth (0.4 – 2.5 THz) is limited by the transmission characteristics of the quartz substrate. The THz waveform is sampled in the time-domain using a time-delayed portion of the fundamental that is detected using a standard balanced photodetection scheme. For photoconductivity measurements, we measure the change in the waveform with and without optical pumping. For photoexcitation of our polymer films, we use a 1400 nm pump pulse that is generated using an optical parametric amplifier. The full transient electric field is measured at several time delays relative to the arrival of the pump pulse. Alternatively, the full decay kinetics are measured at the peak of the transient waveform. The spot sizes of the robe and pump pulses are 1 mm and 1.5 mm, respectively.

3.5. Photoconductor Fabrication and Device Characterization

Synthetic quartz-coated glass substrates were sequentially cleaned using 2% Hellmanex detergent in DI water, DI water, acetone, and isopropyl alcohol for 10 min using sonication, followed by drying in an oven. The substrates were then treated in a UV-ozone cleaner for 30 minutes. Gold electrodes (60 nm) were thermally evaporated at 1 × 10^{-7} torr using a shadow mask. The electrodes were separated by a 60 μm × 1 mm spacing. The polymer was dissolved in chlorobenzene at 100 °C to make a 10 mg ml^{-1} solution in an N_2 atmosphere. The 10 mg ml^{-1} polymer solution was drop cast onto the substrate and allowed to dry. Next, another shadow mask was placed over the center of the substrate, followed by deposition of 100 nm alumina at 1 × 10^{-7} torr over the center of the detector using electron beam evaporation.

The detector chip was mounted in a ceramic LCC. Ultrasonically bonded, gold wires were connected the gold contact pads on the chip to the LCC leads. The LCC was then mounted in a custom-designed, printed circuit board (PCB). For responsivity measurements, the detector was illuminated with a cavity blackbody radiator (Santa Barbara Infrared Inc.) at 500 °C equipped with a mechanical chopper. The PCB was connected by way of triaxial cables to a low-noise current preamplifier (SR570, Stanford Research Systems) and lock-in amplifier (SR830, Stanford Research Systems). Data acquisition was computer-controlled using a custom written program. A Spectra-Physics Solstice femtosecond laser (1-kHz repetition rate, 90 fs pulse width, 800 nm) was used to pump a TOPAS optical parametric amplifier to produce a wavelength of 1550 nm with a pulse width of 150 fs and a repetition rate of 1 kHz. A pair of linear polarizers was used to control the laser power. The photocurrent was routed by way of triaxial cables to an SR570 current preamplifier and voltage amplifier (SR560, Stanford Research Systems) and then finally to a 300 MHz digitizing oscilloscope (Tektronix DPO3034). Electrical phase angle measurements were made using an Agilent E4980 LCR meter.

The responsivity of fully packaged detectors $\Re(\lambda_c, f)$ was calculated using equation S3.[5] This is a standard formula used to calculate detector responsivity using a blackbody source chopped at frequency *f* and is essentially photocurrent *i* divided by the optical power emitted by the blackbody impinging the detector's active area

$$\Re(\lambda_c, f) = \frac{i}{\left(\frac{hc}{\lambda_c}\right)\left[\int_0^{\lambda_c} \frac{2c\pi}{\lambda^4\left(e^{hc/\lambda kT}-1\right)} d\lambda\right]\left(\frac{A_s A_d}{\pi r^2}\right) t F_F} \quad \text{(Eq. S3)}$$

where *h* is Planck's constant, *c* is the speed of light, λ_c is the detector cutoff wavelength, *k* is the Boltzmann constant, *T* is the temperature, A_s is the source area, A_d is the detector area, *t* is the aperture transmittance, *r* is the source-to-detector distance, and F_F is the chopper form factor for conversion of peak-to-peak to rms. The first term of the denominator in brackets is recognizable as photon energy at the detector cutoff wavelength λ_c. The second term represents the exitance of a blackbody in units of photons sr^{-1} s^{-1} cm^{-2} μm^{-1}. Finally, the third term is a testing-setup-specific factor describing the source–detector testing geometry. For reference, the blackbody power incident on the detector active area, calculated using the denominator of (Eq. S3), is 2.31 nW without a spectral filter (broadband). Measurable photocurrent within these spectral regions is consistent with the high *D** obtained and is comparable to other highly optimized

detector technologies. Beyond 9 μm, there was an insufficiently small signal-to-noise ratio that prevented differentiation between photocurrent from in-band noise transduced by the lock-in amplifier.

The photoconductor was also measured using a 1550 nm laser (LQD1550-05E, Newport). The laser was adjusted using a waveform controller (4064B, B&K Precision) and the laser intensity was controlled by a variable neutral-density filter (NDC-50S-3, ThorLabs) and measured by an optical sensor (PM400, ThorLabs) using a sensor head (S401C, ThorLabs). For responsivity ($\Re$) measurements, the laser pulse is set to 100 mHz frequency and 5 V peak-to-peak amplitude. A 5 V bias voltage with 0.1 A compliance was applied, which gives a $\Re$ of 2.07×10^{-2} A W^{-1} (Figure S16a). The detectivity is given by $D^* = \Re\sqrt{A}/S_n$ where A is detector area in a unit of cm^2 and S_n is noise in a unit of A $Hz^{-1/2}$. The noise was calculated using $S_n = \sqrt{4kT/R'}$ where k is the Boltzmann constant in a unit of m^2 kg s^{-2} K^{-1}, T is operation temperature in a unit of K, and R' is resistance of the device in a unit of Ω, obtained from the *I-V* curve of the dark current (Figure S16b). These measurements give $D^* = 3.69 \times 10^9$ Jones.

3.6. Grazing-Incidence Wide-Angle X-ray Scattering (GIWAXS)

GIWAXS samples were prepared by spin-coating a film from chlorobenzene onto a Si wafer (as previously described). The spin coated samples were measured at beamline 11-3 at the Stanford Synchrotron Radiation Lightsource (SSRL), in a helium-filled chamber, at an incidence angle of 0.12°, with a Rayonix Mar CCD225 detector. The sample-to-detector distance was 300 mm. An X-ray beam wavelength of 0.9758 Å and exposure time of 400 s was used. The data were processed using WaveMetrics Igor Pro with the Nika script and WAXStools software.

3.7. Electron Paramagnetic Resonance Spectroscopy

Room temperature continuous-wave EPR spectra were recorded on a Bruker EMXmicro EPR spectrometer operating in the X-band. Solid-state samples were loaded into 4 mm quartz tubes and evacuated to 40 mbar for 12 hours before flame sealing under vacuum. Spin concentration was obtained by comparing solid-state samples against a 2,2-diphenyl-1-picrylhydrazyl standard with a known spin concentration. Variable-temperature measurements were performed on a Bruker E540 or E680 EPR spectrometer operating at the X-band. The EPR signal intensity from 32 to 5 K was utilized to extract the singlet–triplet energy splitting (ΔE_{ST}) by fitting the data to the Bleaney–Bowers equation[6] (S4):

$$I_{\mathrm{EPR}} = \frac{C}{T}\frac{3e^{-2J/k_{\mathrm{B}}T}}{1+3e^{-2J/k_{\mathrm{B}}T}} \qquad \text{(Eq. S4)}$$

where C is a constant, k_B is the Boltzmann constant, J is the intramolecular exchange coupling constant, and $2J$ is ΔE_{ST}. From the fit parameters, a $\Delta E_{ST} = 1.53 \times 10^{-2}$ kcal mol^{-1} was obtained.

3.8. Superconducting Quantum Interference Device (SQUID) Magnetometry

Magnetometry data was collected using the Quantum Design MPMS3 SQUID-VSM. The sample was loaded into a polycarbonate capsule and placed in a standard straw sample holder. After the measurements, the background signal of the sample holder and capsule were recorded and subtracted from the original signal. For the magnetization as a function of field isotherms, the magnetic moment was recorded after allowing the sample to reach thermal equilibrium at the measurement temperature, within the range -70,000 Oe ≤ H ≤ 70,000 Oe, beginning from the high field, ramping down incrementally, and allowing the field to stabilize at each step before measuring. The lowest-temperature data was then fit to the Brillouin function for the determination of the ground-state net spin. The Brillouin function is defined as

$$M = M_0\left[\frac{2S+1}{2S}\coth\left(\frac{2S+1}{2S}\frac{gS\mu_{\mathrm{B}}H}{kT}\right)-\frac{1}{2S}\coth\left(\frac{1}{2S}\frac{gS\mu_{\mathrm{B}}H}{kT}\right)\right]$$

where H is the applied magnetic field, T is the temperature, g is the g-factor, k_B is the Boltzmann constant, M_0 is the saturation magnetization, μ_B is the Bohr magneton and S, the spin quantum number, is the fitting parameter. From the fit, we find $S = 0.98$ (Figure S4), consistent with a triplet ground state.

3.9. Quantum Chemical Calculations

In recent years, computational tools have been utilized to describe the open-shell character of small molecular and polymeric systems based on their electronic properties. The contributions of strong π-correlations between unpaired electrons to the ground state electronic structure can be described in terms of the singlet-triplet gap (ΔE_{ST}), diradical character index (y), topological arrangement of unpaired electrons, spin density distribution between the α- and β-frontier molecular orbitals (FMOs), electrostatic potential surface, and bond length alternation (BLA). DFT calculations on the model oligomer units were performed by progressively increasing the size of the π-system from n = 1 to 5 using the Gaussian 16 software package. Molecular geometries for the electronic ground state (S_0) and the triplet state of these oligomers were optimized in the gas phase using density functional theory (DFT) with Becke's three-parameter functional B3LYP and 6-31G** basis set. Alkyl side chains were truncated with methyl ($-CH_3$) groups. Broken-symmetry (BS) singlet state calculations were started with a restricted wave function. The diradical index (y) was calculated using both HONO and LUNO occupation numbers and Yamaguchi's formula (Table S2). The spin locations were predicted from the natural spin densities of Kohn-Sham molecular orbitals (MO). Molecular electrostatic potential (MESP) surface plots were obtained at the UB3LYP/6-311G** level of theory. The conductor-like polarizable continuum (C-PCM) model was employed to simulate the TDDFT results in a chlorobenzene solvent medium at the UB3LYP/6-311G** level of theory. The optimized ground state pentamer (n = 5) geometry showed tetraradical character (i.e., y_0 = 0.268 at the central BBT acceptor unit and y_1 = 0.161 at the flanked thiophene segments) (Figure S7 and Table S2). Nonetheless, the electron density was distributed over the conjugated skeleton with major localization on the BBT acceptor units (Figure S8-S12). The intramolecular interactions of the BBT unit with flanked thiopehene units strengthened the rigidity of the π-framework, revealed from MESP analysis (Figure S13). The longer wavelength transitions noted from the closer energy levels of α- and β- orbitals are consistent with spectral broadening in the IR (Figure S14 and Table S3).

4. Supplemental Figures S1–S16

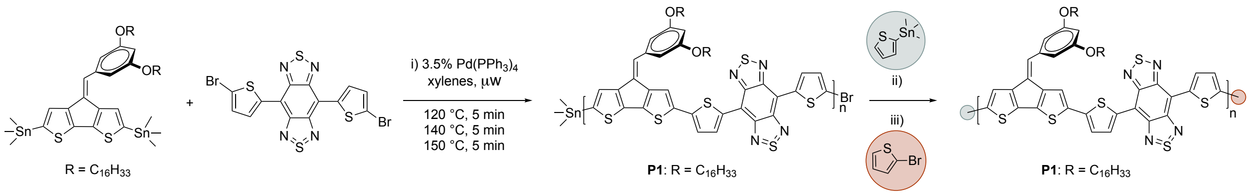

Figure S1. Synthesis of the polymer using a microwave-mediated Stille cross-coupling copolymerization.

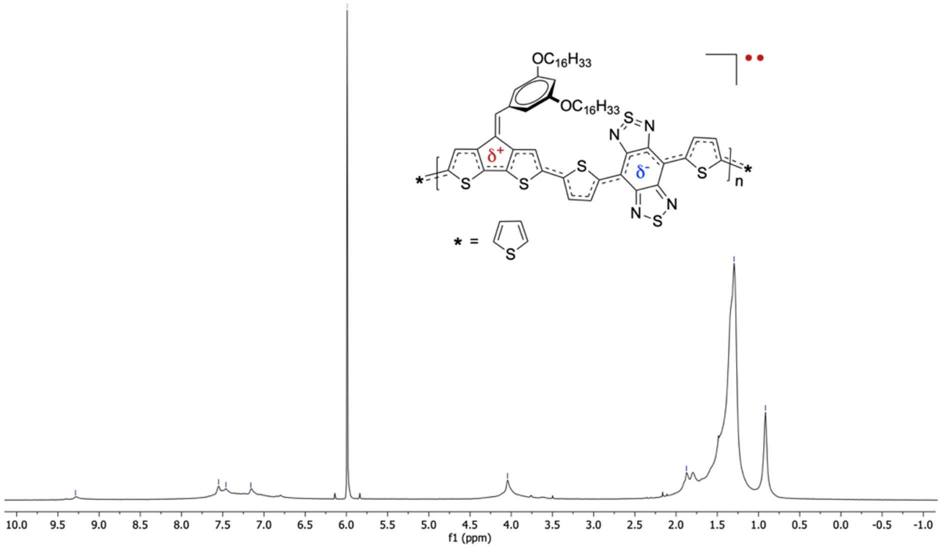

Figure S2. ^1H NMR spectrum of the polymer. ^1H NMR (600 MHz, 1,1,2,2-tetrachloroethane-d_2, 413 K) of poly((4-(3,5-bis(hexadecyloxy)benzylidene)-4*H*-cyclopenta[2,1-*b*:3,4-*b'*]dithiophene-2,6-diyl)-*alt*-4,7-bis(5-thiophen-2-yl)-2$\lambda^4\delta^2$-benzo[1,2-*c*;4,5-*c'*]bis[1,2,5]thiadiazole).

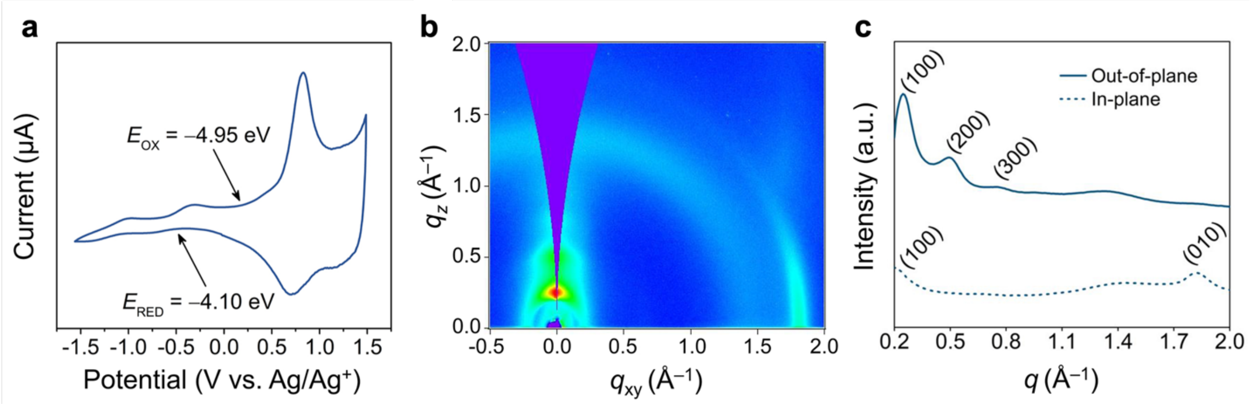

Figure S3. Solid-state properties of polymer thin films. (a) Cyclic voltammetry indicates an onset of oxidation at −4.95 eV and an onset of reduction at −4.10 eV. (b) Two-dimensional line cuts of the integrated in-plane and out-of-plane GIWAXS profiles. (c) The corresponding one-dimensional GIWAXS profile.

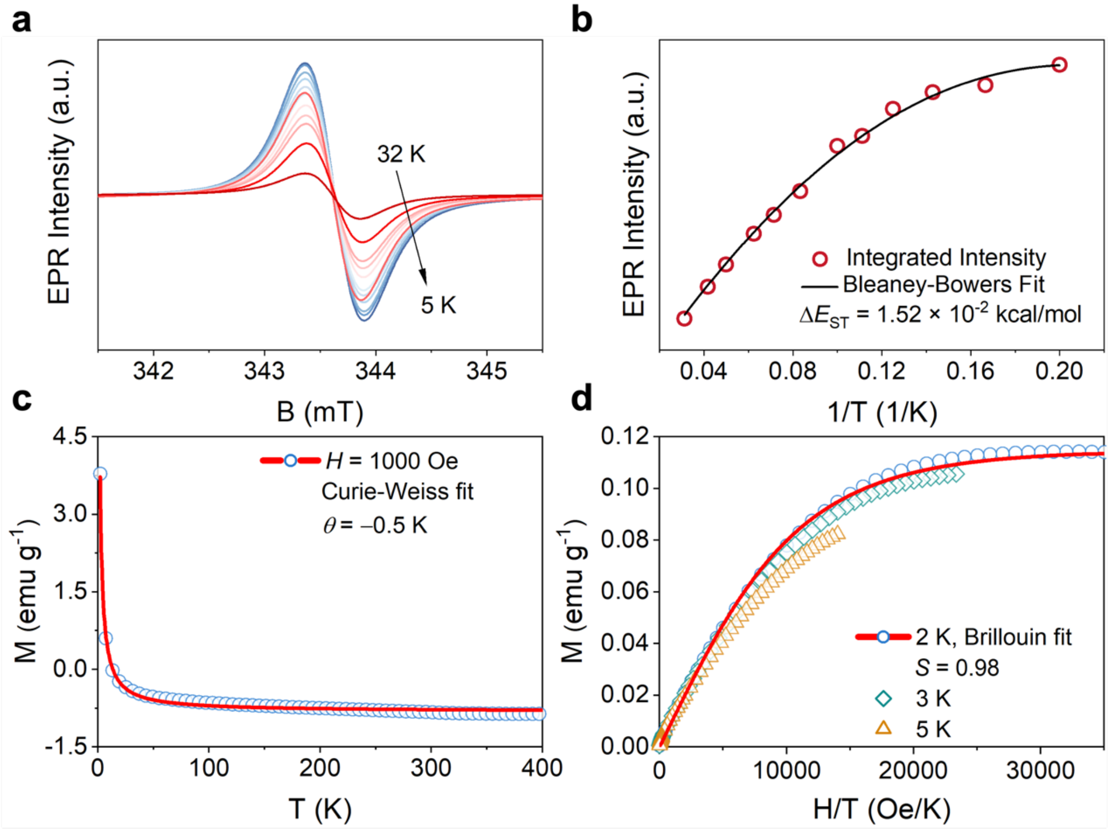

Figure S4. (a) EPR (X-band) spectra of the polymer from 32 K to 5 K. (b) Temperature-dependent fit to the Bleaney-Bowers equation with ΔE_{ST} of 1.52×10^{-2} kcal mol^{-1}. (c) Temperature dependence of the magnetization of the polymer measured over a range from 2-400 K under an applied field of 1000 Oe. (d) The field-dependent magnetization at 2 K fit to the paramagnetic Brillouin function to give S = 0.98.

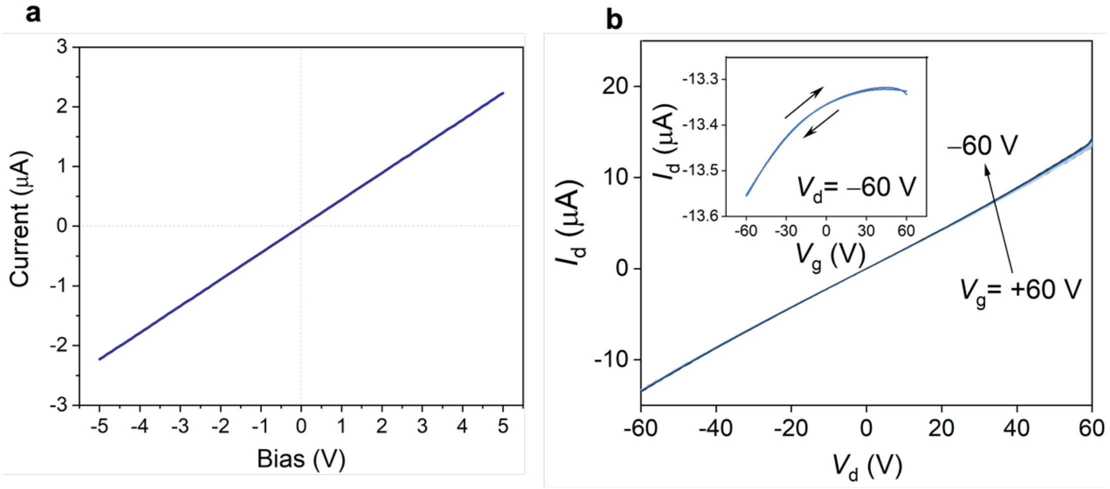

Figure S5. Current-voltage characteristics of drop-cast films and output characteristics of spin-coated films.

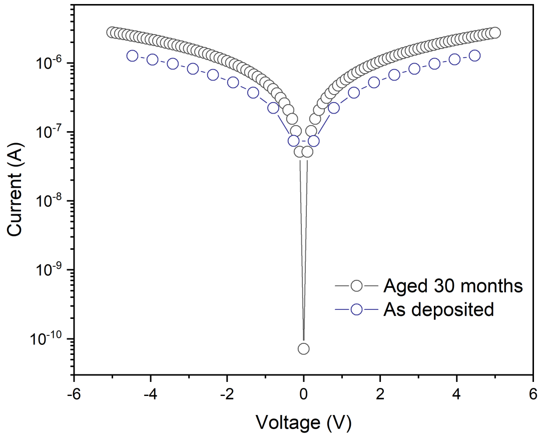

Figure S6. Stability study of the device. Measurement of the device showed no discernable changes over a period of greater than one year.

Table S1. Comparison of Photoconductive IR detectors Under Blackbody Illumination. Room temperature (RT) is defined here as 20-25 ºC.

Material	Wavelength Range (µm)	Blackbody Temperature (ºC)	Operating Temperature (ºC)	*R* (A/W)	*D** (Jones)	Ref.
Our Work	0.5-16.5	500	RT	1.78×10^{-2}	2.93×10^{9}	-
PbS	1-3.5	500	RT	-	7.7×10^{8}	Teledyne Judson Technologies
PbS	1-3.5	500	-30	-	2.3×10^{9}	Teledyne Judson Technologies
PbSe	2-6	500	RT	-	5.5×10^{8}	Teledyne Judson Technologies
PbSe	2-6	500	-65	-	3.2×10^{9}	Teledyne Judson Technologies
$InAs_{0.91}Sb_{0.09}$	1-4	727	RT	0.27	2.4×10^{7}	[7]
$InAs_{0.91}Sb_{0.09}$	1-4	727	-196	15.82	6.1×10^{9}	[7]
HgCdTe	2.2-10.6	800	RT	-	1×10^{9}	VIGO Photonics
Colloidal PbSe nanocrystals	3-5	900	RT	0.64	8.36×10^{8}	[8]
Te nanowire	0.5-2.5	927	RT	2.53	4.68×10^{8}	[9]
Te nanosheet	0.5-2.5	927	RT	1.59	2.06×10^{7}	[9]
HgTe QD	3-5	1200	-143	0.2	2×10^{9}	[10]

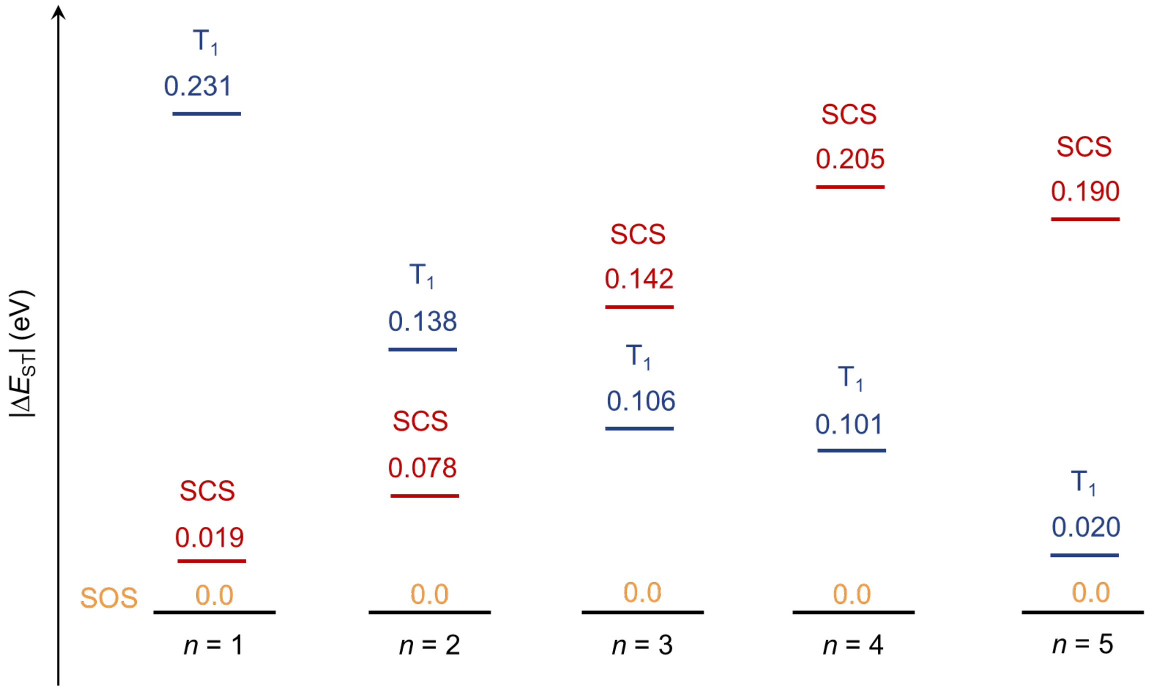

Figure S7. Absolute value of ΔE_{ST} of the polymer as a function of repeat units *(n)* showing the change in the relative energies of the various spin states with reference to singlet open-shell (SOS) state. T1: Triplet state; SCS: singlet closed shell.

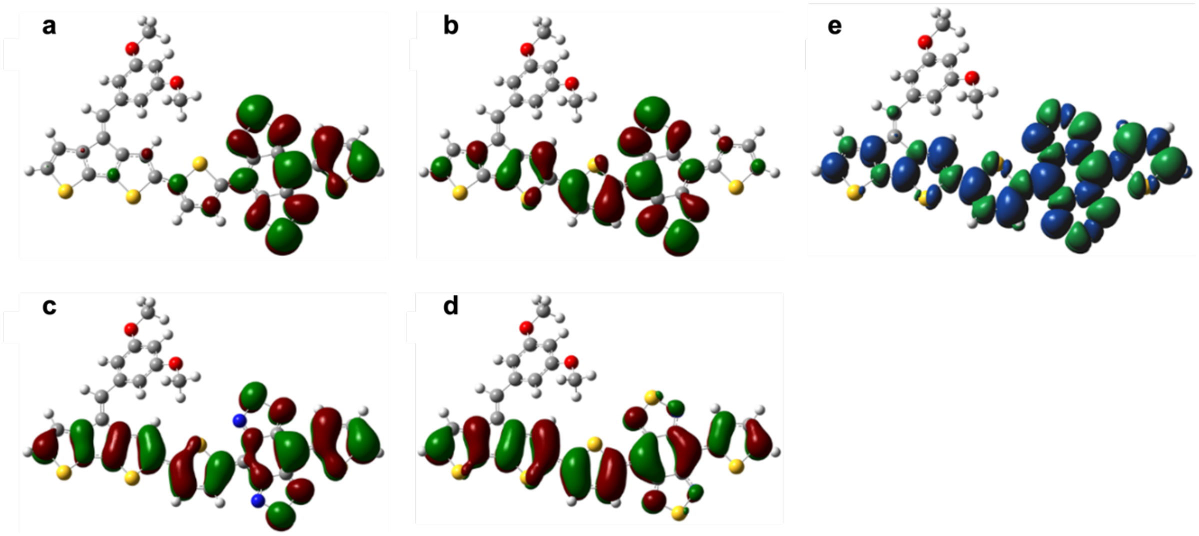

Figure S8. Optimized ground state geometric structures for the single repeat unit (*n* = 1) of the polymer and pictorial representations of the frontier MOs and spin density distribution. (a) α-SUMO and (b) β-SUMO, (c) α-SOMO and (d**)** β-SOMO, and (e) Spin density distribution of the open-shell singlet. The green and red surfaces represent positive and negative signs of the MO at isovalue = 0.02 au, respectively. The blue and green surfaces represent positive and negative contributions of the spin density at an isovalue = 0.0004 au. Color codes for the atoms are: gray for C, blue for N and yellow for S.

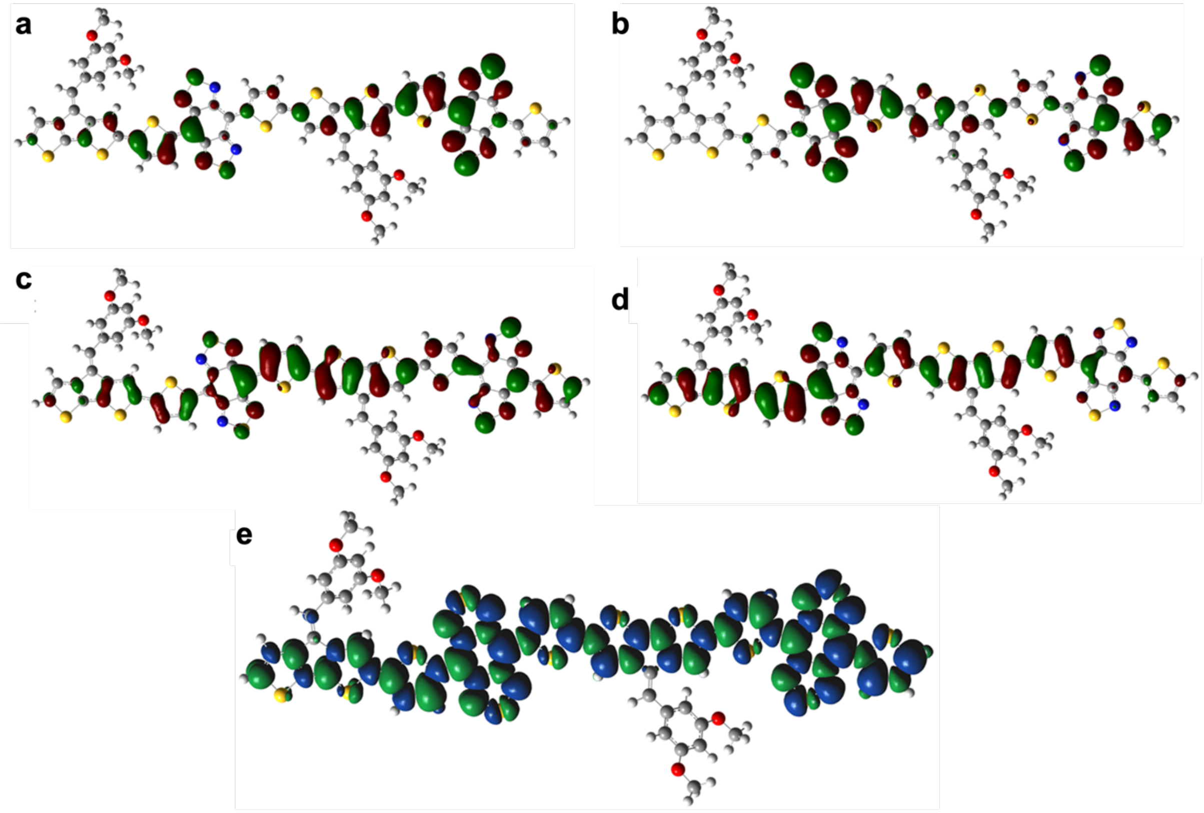

Figure S9. Optimized ground state geometric structures for the dimer (n = 2) of the polymer and pictorial representations of the frontier MOs and spin density distribution. (a) α-SUMO and (b) β-SUMO, (c) α-SOMO and (d**)** β-SOMO, and (e) Spin density distribution of the open-shell singlet. The green and red surfaces represent positive and negative signs of the MO at isovalue = 0.02 au, respectively. The blue and green surfaces represent positive and negative contributions of the spin density at an isovalue = 0.0004 au. Color codes for the atoms are: gray for C, blue for N and yellow for S.

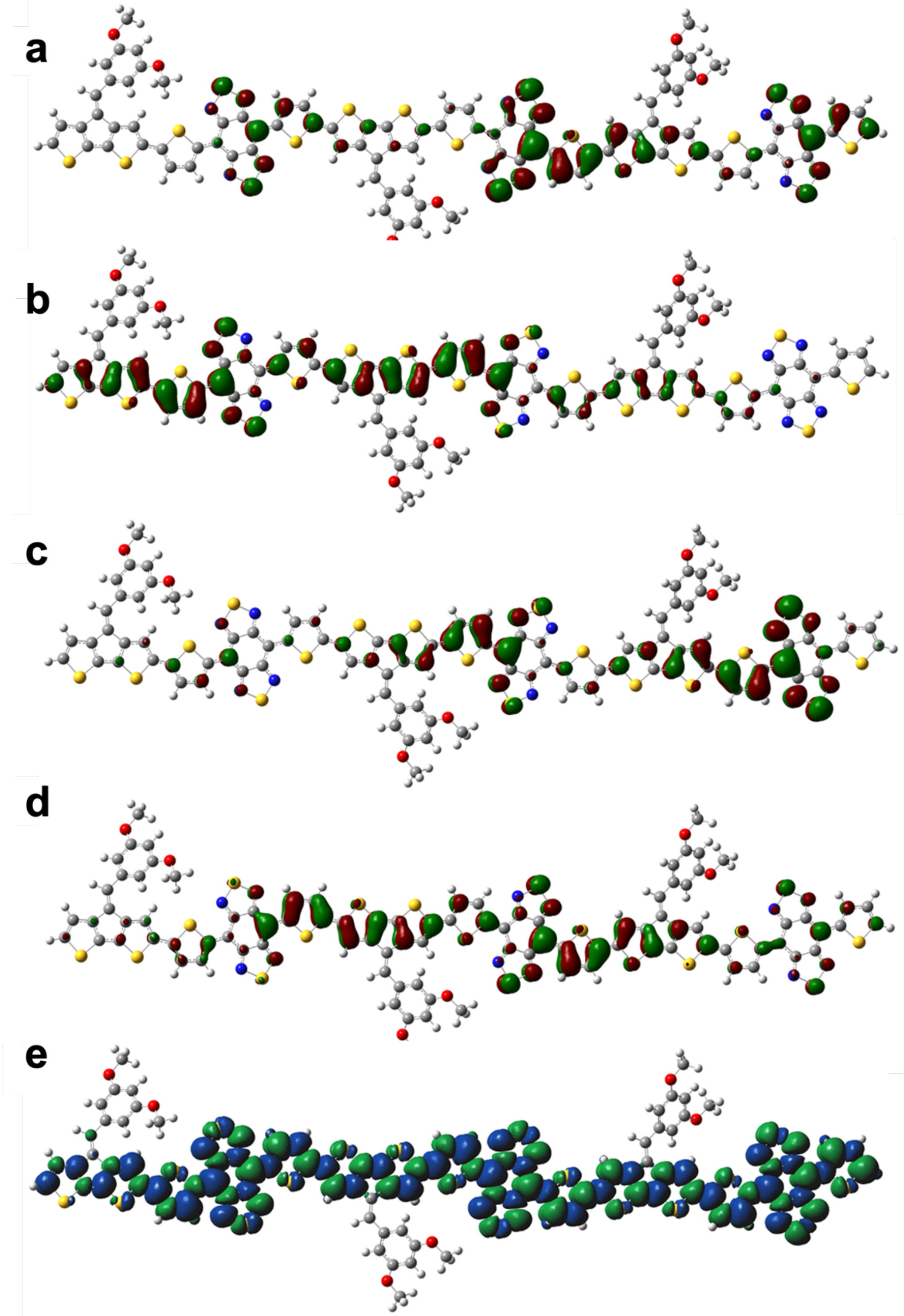

Figure S10. Optimized ground state geometric structures for the trimer (n = 3) of the polymer and pictorial representations of the frontier MOs and spin density distribution. (a) α-SUMO and (b) β-SUMO, (c) α-SOMO and (d**)** β-SOMO, and (e) Spin density distribution of the open-shell singlet. The green and red surfaces represent positive and negative signs of the MO at isovalue = 0.02 au, respectively. The blue and green surfaces represent positive and negative contributions of the spin density at an isovalue = 0.0004 au. Color codes for the atoms are: gray for C, blue for N and yellow for S.

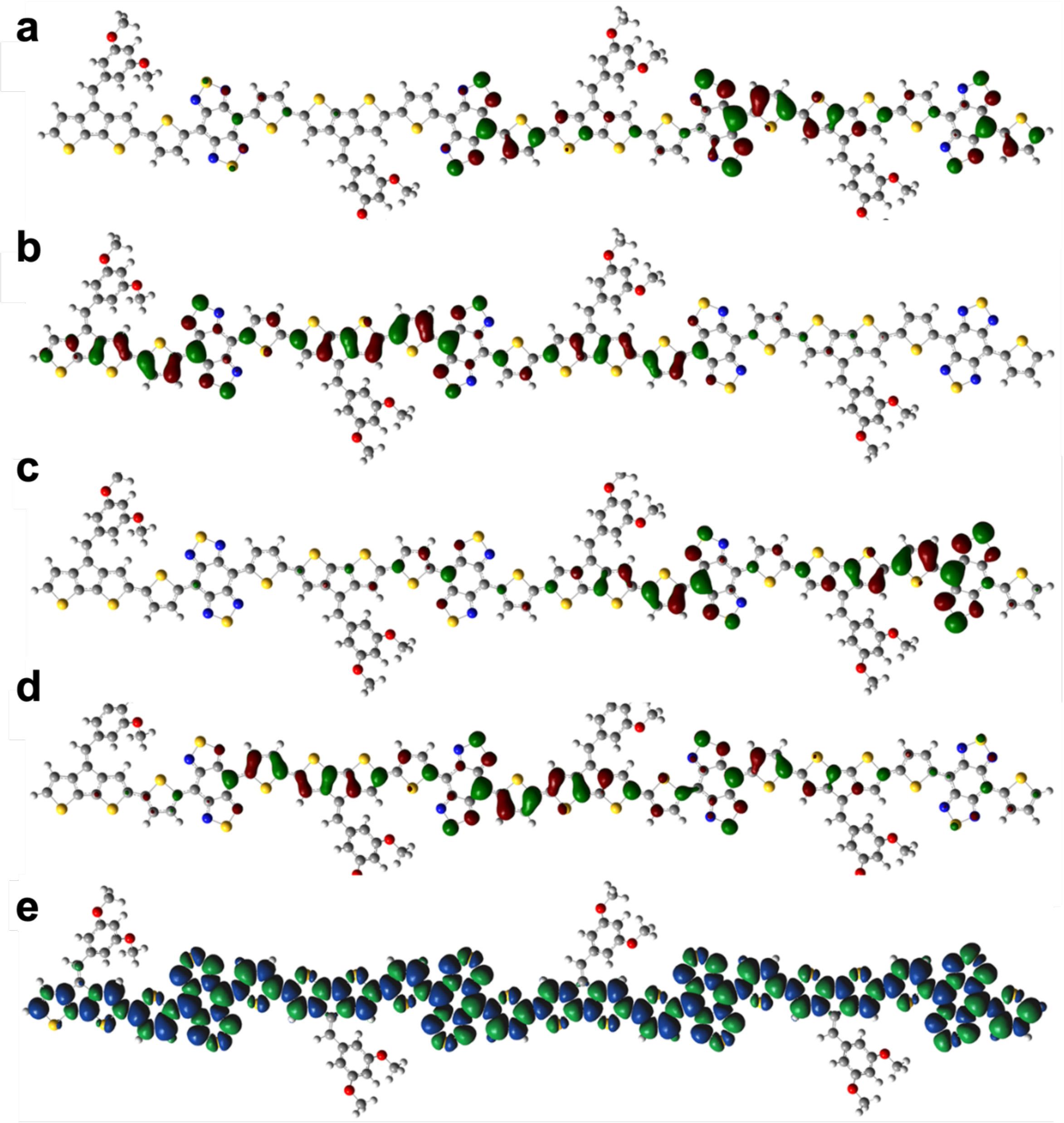

Figure S11. Optimized ground state geometric structures for the tetramer (*n* = 4) of the polymer and pictorial representations of the frontier MOs and spin density distribution. (a) α-SUMO and (b) β-SUMO, (c) α-SOMO and (d**)** β-SOMO, and (e) Spin density distribution of the open-shell singlet. The green and red surfaces represent positive and negative signs of the MO at isovalue = 0.02 au, respectively. The blue and green surfaces represent positive and negative contributions of the spin density at an isovalue = 0.0004 au. Color codes for the atoms are: gray for C, blue for N and yellow for S.

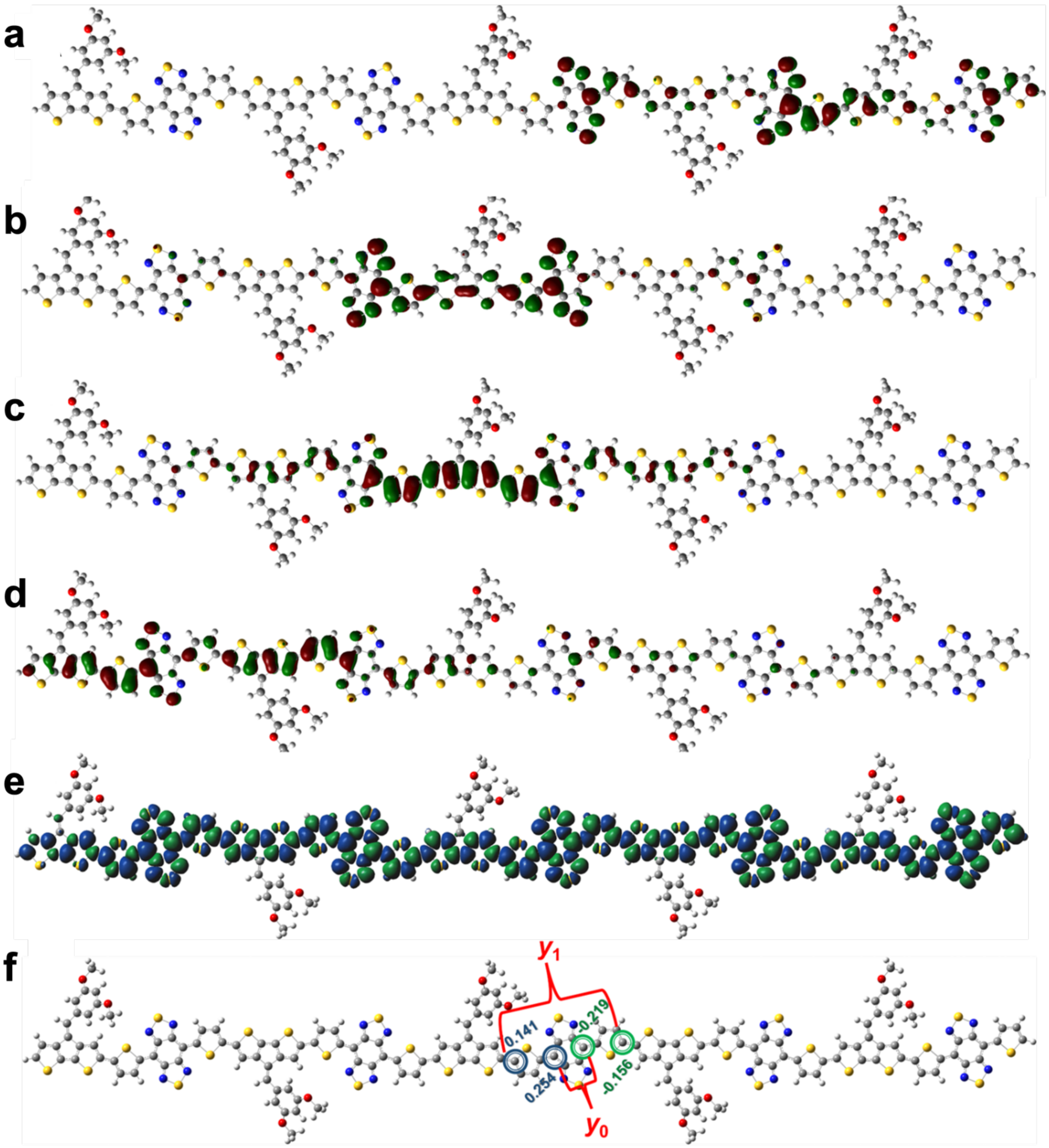

Figure S12. Optimized ground state geometric structures for the pentamer (n = 5) of the polymer and pictorial representations of the frontier MOs and spin density distribution. (a) α-SUMO and (b) β-SUMO, (c) α-SOMO and (d) β-SOMO, and (e) Spin density distribution. (f) Spin density population values corresponding to the diradical (y_0) and tetraradical (y_1) of the open-shell singlet. The green and red surfaces represent positive and negative signs of the MO at isovalue = 0.02 au, respectively. The blue and green surfaces represent positive and negative contributions of the spin density at an isovalue = 0.0004 au. Color codes for the atoms are: gray for C, blue for N and yellow for S.

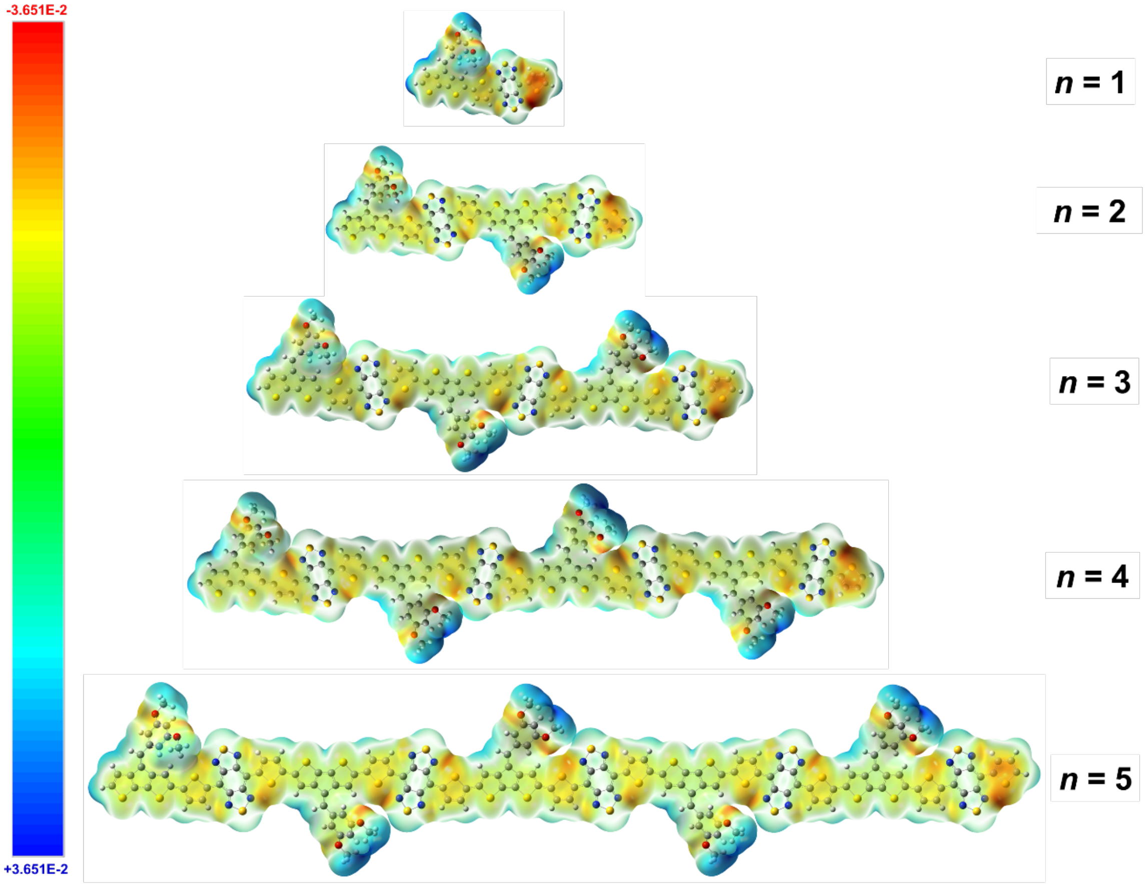

Figure S13. Molecular electrostatic potential surface plots (MESP) for the *n* = 1, 2, 3, 4, and 5 repeated units of the polymer.

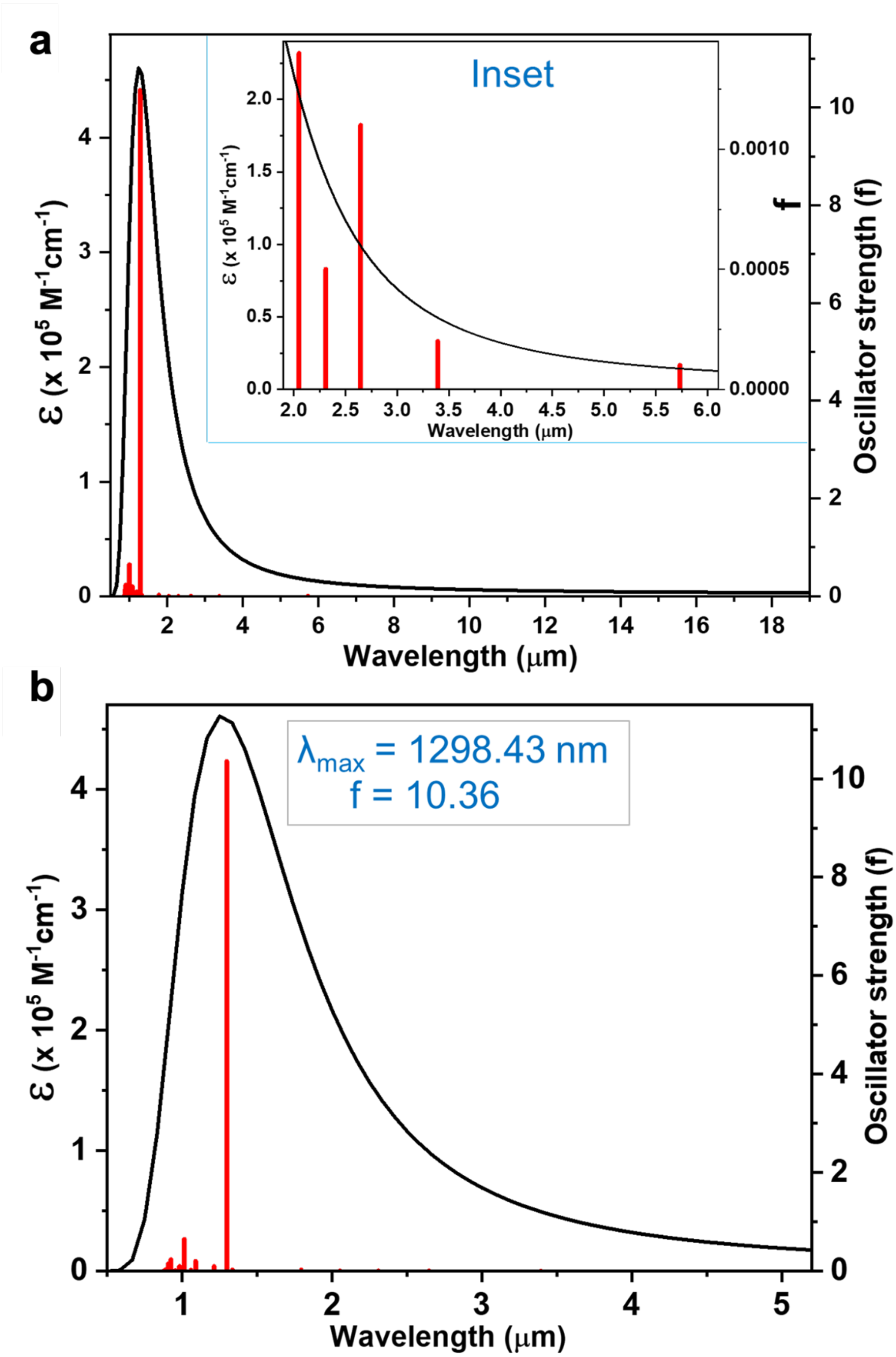

Figure S14. (a) TDDFT simulated spectra of the pentamer showing the major transitions involved; Inset displays the magnified spectral wavelength at the SWIR region. (b) Absorption maxima, molar extinction coefficients (ε) and oscillator strength (f) obtained from B3LYP/6-311g(d, p)/C-PCM(C_6H_5Cl) level of theory.

Table S2. Selected electronic properties of oligomers as a function of the number of repeat units.

n^a	$\Delta E_{ST}{}^b$ (eV)	nHONO^c	nLUNO^c	y_d	$S_{degen}{}^e$ (y_0)	$S_{degen}{}^e$ (y_1)	SOS-SCS (eV)	$\mu_g{}^e$ (Debye)
**1**	0.231	1.77723	0.22277	0.031	0.0346	-	0.019	3.6
**2**	0.138	1.54356	0.45644	0.161	0.0174	-	0.078	3.2
**3**	0.106	1.41305	0.58695	0.294	0.0362	-	0.142	2.9
	-	1.72965	0.27035	0.048	-	0.0346		
**4**	0.101	1.33638	0.66362	0.396	0.0384	-	0.205	2.5
	-	1.6403	0.3597	0.092	-	0.0321		
**5**	0.020	1.43521	0.56479	0.268	0.0376	-	0.190	3.3
	-	1.54291	0.45709	0.161	-	0.0332		

[a]Number of repeat units (n) for the calculated oligomers. [b]Singlet^{BS}-triplet energy gap. [c]Natural orbital occupancies, [d]diradical character index di- (y_0) and tetra-radical (y_1) calculated from Yamaguchi's formula. [e]degeneracy of the singlet (energy difference between the two NBO e^-s) computed for the di- (y_0) and tetra-radical (y_1) formation and [f]ground state dipole moment as determined at the UB3LYP/6-31G** level of theory. Natural orbital occupancies (HONO, LUNO) and y are unitless quantities. SCS: singlet closed shell; SOS: singlet open shell.

Table S3: Computed excitation energies (nm) with electron volt (eV) values in parenthesis, oscillator strength (f), major and minor transitions involved upon photoexcitation obtained from the TDDFT simulation (Figure S12) using B3LYP/6-311g(d, p)/C-PCM(C_6H_5Cl) level of theory.

B3LYP	λ_{max} (nm)	f	Major and Minor transitions
**S_0-S_1**	5732.66 (0.2163)	0.0001	*H-1(A)->LUMO(A) (18%), HOMO(A)->LUMO(A) (14%), HOMO(B)->L+1(B) (28%)*<br>*H-7(A)->L+6(A) (4%), H-6(A)->L+5(A) (2%), H-5(A)->L+4(A) (2%), H-5(A)->L+6(A) (2%), H-4(A)->L+3(A) (3%), H-4(A)->L+5(A) (2%), H-3(A)->LUMO(A) (2%), H-3(A)->L+2(A) (4%), H-3(A)->L+4(A) (2%), H-2(A)->LUMO(A) (5%), H-2(A)->L+1(A) (5%), H-2(A)->L+3(A) (2%), H-1(A)->L+2(A) (4%), HOMO(A)->L+1(A) (4%), H-6(B)->L+6(B) (2%), H-5(B)->L+6(B) (3%), H-3(B)->L+4(B) (2%), H-2(B)->L+1(B) (3%), H-2(B)->L+3(B) (2%), H-2(B)->L+4(B) (2%), H-1(B)->L+2(B) (4%), H-1(B)->L+3(B) (3%), H-1(B)->L+4(B) (2%), HOMO(B)->LUMO(B) (5%), HOMO(B)->L+2(B) (7%), HOMO(B)->L+3(B) (3%)*
**S_0-S_2**	3392.26 (0.3655)	0.0002	*H-1(B)->L+1(B) (10%)*<br>*H-6(A)->L+6(A) (2%), H-4(A)->L+6(A) (3%), H-3(A)->LUMO(A) (6%), H-3(A)->L+5(A) (2%), H-2(A)->LUMO(A) (4%), H-2(A)->L+4(A) (2%), H-1(A)->LUMO(A) (5%), H-1(A)->L+1(A) (4%), H-1(A)->L+2(A) (2%), H-1(A)->L+3(A) (2%), H-1(A)->L+4(A) (2%), HOMO(A)->L+1(A) (8%), HOMO(A)->L+2(A) (5%), HOMO(A)->L+4(A) (2%), H-4(B)->L+6(B) (2%), H-*

			3(B)->L+1(B) (3%), H-2(B)->L+6(B) (2%), H-1(B)->LUMO(B) (4%), H-1(B)->L+4(B) (2%), HOMO(B)->L+1(B) (2%), HOMO(B)->L+2(B) (6%), HOMO(B)->L+3(B) (9%), HOMO(B)->L+4(B) (3%)
**S_0-S_3**	2647.50 (0.4683)	0.0011	*No major transitions involved*
			H-4(A)->LUMO(A) (5%), H-3(A)->L+1(A) (3%), H-3(A)->L+6(A) (4%), H-2(A)->LUMO(A) (2%), H-2(A)->L+1(A) (2%), H-2(A)->L+5(A) (2%), H-1(A)->L+1(A) (5%), H-1(A)->L+3(A) (4%), H-1(A)->L+5(A) (3%), H-1(A)->L+6(A) (2%), HOMO(A)->L+2(A) (4%), HOMO(A)->L+3(A) (5%), HOMO(A)->L+5(A) (3%), H-4(B)->L+1(B) (2%), H-3(B)->L+6(B) (2%), H-2(B)->LUMO(B) (2%), H-2(B)->L+1(B) (4%), H-1(B)->LUMO(B) (2%), H-1(B)->L+1(B) (2%), H-1(B)->L+2(B) (4%), H-1(B)->L+3(B) (4%), H-1(B)->L+6(B) (3%), HOMO(B)->L+3(B) (2%), HOMO(B)->L+4(B) (8%), HOMO(B)->L+5(B) (4%), HOMO(B)->L+6(B) (2%)
**S_0-S_4**	2310.65 (0.5366)	0.0005	*HOMO(B)->L+6(B) (11%)*
			H-7(A)->LUMO(A) (2%), H-5(A)->LUMO(A) (2%), H-4(A)->L+1(A) (3%), H-3(A)->L+2(A) (2%), H-3(A)->L+3(A) (2%), H-3(A)->L+5(A) (2%), H-2(A)->L+3(A) (2%), H-2(A)->L+6(A) (7%), H-1(A)->L+4(A) (2%), H-1(A)->L+6(A) (3%), HOMO(A)->L+3(A) (2%), HOMO(A)->L+4(A) (4%), HOMO(A)->L+6(A) (8%), H-5(B)->L+1(B) (3%), H-3(B)->L+1(B) (2%), H-3(B)->L+5(B) (2%), H-2(B)->L+2(B) (2%), H-2(B)->L+6(B) (2%), H-1(B)->L+4(B) (4%), H-1(B)->L+5(B) (2%), HOMO(B)->L+5(B) (5%)
**S_0-S_5**	2053.58 (0.6037)	0.0014	*No major transitions involved*
			H-6(A)->LUMO(A) (2%), H-5(A)->L+1(A) (2%), H-4(A)->L+4(A) (2%), H-2(A)->L+1(A) (3%), H-2(A)->L+4(A) (4%), H-2(A)->L+5(A) (2%), H-1(A)->L+1(A) (2%), H-1(A)->L+2(A) (2%), H-1(A)->L+4(A) (4%), H-1(A)->L+5(A) (9%), H-1(A)->L+6(A) (4%), HOMO(A)->L+5(A) (2%), H-3(B)->LUMO(B) (2%), H-3(B)->L+3(B) (2%), H-2(B)->LUMO(B) (3%), H-2(B)->L+4(B) (4%), H-2(B)->L+5(B) (2%), H-1(B)->LUMO(B) (2%), H-1(B)->L+4(B) (2%), H-1(B)->L+5(B) (7%), H-1(B)->L+6(B) (3%), HOMO(B)->L+5(B) (3%), HOMO(B)->L+6(B) (6%)
**S_0-S_6**	1796.04 (0.6903)	0.0240	*HOMO(A)->L+4(A) (10%), HOMO(A)->L+5(A) (11%)*
			H-1(A)->L+3(A) (2%), H-1(A)->L+4(A) (3%), H-1(A)->L+5(A) (2%), HOMO(A)->L+2(A) (3%), HOMO(A)->L+3(A) (6%), HOMO(A)->L+6(A) (8%), H-3(B)->L+1(B) (2%), H-3(B)->L+2(B) (3%), H-3(B)->L+7(B) (2%), H-2(B)->L+2(B) (3%), H-2(B)->L+3(B) (2%), H-2(B)->L+7(B) (4%), H-1(B)->L+2(B) (2%), H-1(B)->L+3(B) (2%), H-1(B)->L+7(B) (6%), HOMO(B)->L+7(B) (3%)
**S_0-S_7**	1337.67 (0.9269)	0.0239	*No major transitions involved*
			H-4(A)->L+2(A) (2%), H-3(A)->L+1(A) (3%), H-3(A)->L+2(A) (5%), H-3(A)->L+3(A) (4%), H-2(A)->L+1(A) (4%), H-2(A)->L+2(A) (9%), H-2(A)->L+3(A) (8%), H-2(A)->L+4(A) (3%), H-1(A)->L+1(A) (2%), H-1(A)->L+2(A) (5%), H-1(A)->L+3(A) (5%), H-1(A)->L+4(A) (2%), H-6(B)->LUMO(B) (3%), H-5(B)->LUMO(B) (3%), H-4(B)->LUMO(B) (4%), H-3(B)->LUMO(B) (4%), H-2(B)->LUMO(B) (4%), H-1(B)->LUMO(B) (3%)
**S_0-S_8**	1298.43 (0.9549)	10.3576	*HOMO(A)->LUMO(A) (16%), HOMO(B)->LUMO(B) (14%), HOMO(B)->L+1(B) (18%)*
			H-2(A)->L+1(A) (3%), H-1(A)->LUMO(A) (8%), H-1(A)->L+1(A) (3%), H-1(A)->L+2(A) (2%), HOMO(A)->L+1(A) (5%), H-2(B)->L+2(B) (2%), H-1(B)->LUMO(B) (5%), H-1(B)->L+1(B) (3%), H-1(B)->L+2(B) (4%)

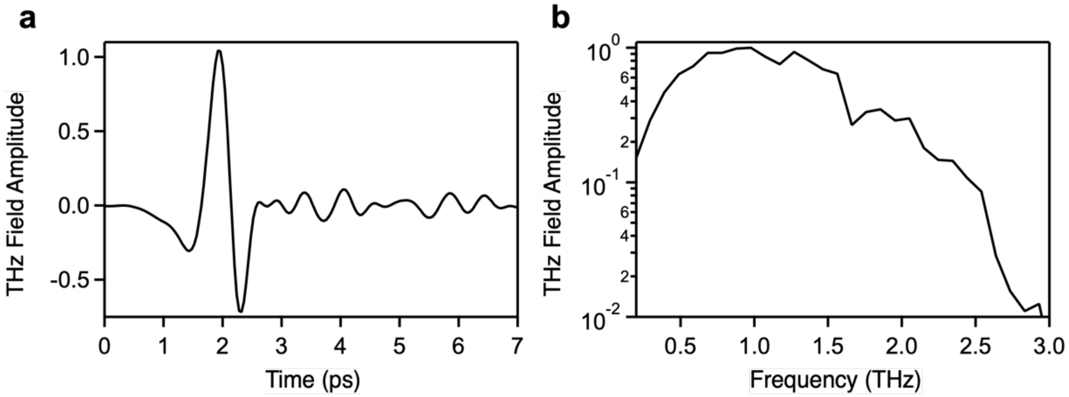

Figure S15. (a) The spectral content of the THz probe pulse is measured in the time domain. (b) A Fourier transform is used to determine the frequency content, which spans 0.4 – 2.5 THz.

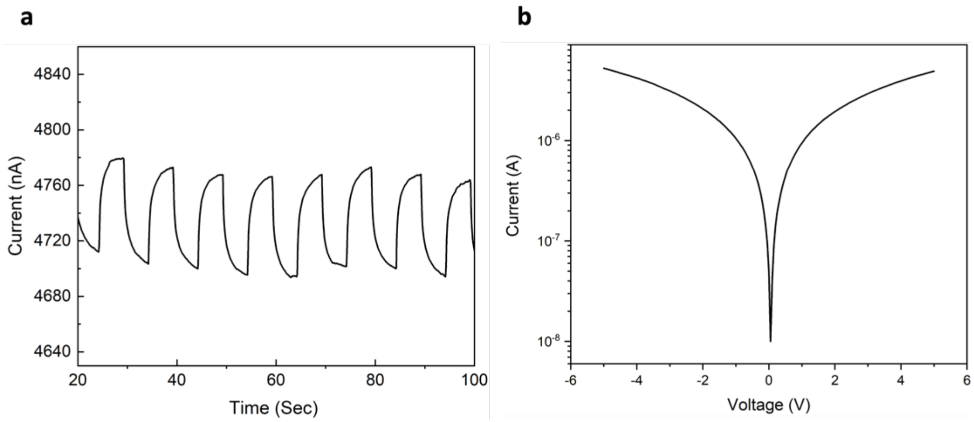

Figure S16. (a) Photocurrent generated using a 1550 nm in ambient conditions. (b) *I-V* curve of the dark current for the device. D^* = 3.69 × 10^9 Jones

References

[1] E. Bundgaard, F. C. Krebs, *Macromolecules* **2006**, *39*, 2823.

[2] Z. Wu, Y. Zhai, W. Yao, N. Eedugurala, S. Zhang, L. Huang, X. Gu, J. D. Azoulay, T. N. Ng, *Adv. Funct. Mater.* **2018**, *28*, 1805738.

[3] M. E. Foster, B. A. Zhang, D. Murtagh, Y. Liu, M. Y. Sfeir, B. M. Wong, J. D. Azoulay, *Macromol. Rapid Commun.* **2014**, *35*, 1461.

[4] L. Huang, N. Eedugurala, A. Benasco, S. Zhang, K. S. Mayer, D. J. Adams, B. Fowler, M. M. Lockart, M. Saghayezhian, H. Tahir, E. R. King, S. Morgan, M. K. Bowman, X. Gu, J. D. Azoulay, *Adv. Funct. Mater.* **2020**, *30*, 1909805.

[5] J. H. Vella, L. Huang, N. Eedugurala, K. S. Mayer, T. N. Ng, J. D. Azoulay, *Sci. Adv.* **2021**, *7*, eabg2418.

[6] M. E. Steelman, D. J. Adams, K. S. Mayer, P. Mahalingavelar, C.-T. Liu, N. Eedugurala, M. Lockart, Y. Wang, X. Gu, M. K. Bowman, J. D. Azoulay, *Adv. Mater.* **2022**, *34*, 2206161.

[7] Y. X. Jinchao Tong, Peinan Ni, Zhengji Xu, Shupeng Qiu, Landobasa Y M Tobing and Dao-Hua Zhang, *Phys. Scr.* **2016**, *91*, 115801.

[8] J. Park, M. M. Al Mahfuz, R. Huebner, D.-K. Ko, *ACS Appl. Electron. Mater.* **2023**, *5*, 2386.
[9] M. Peng, R. Xie, Z. Wang, P. Wang, F. Wang, H. Ge, Y. Wang, F. Zhong, P. Wu, J. Ye, Q. Li, L. Zhang, X. Ge, Y. Ye, Y. Lei, W. Jiang, Z. Hu, F. Wu, X. Zhou, J. Miao, J. Wang, H. Yan, C. Shan, J. Dai, C. Chen, X. Chen, W. Lu, W. Hu, *Sci. Adv.* **2021**, *7*, eabf7358.
[10] S. Keuleyan, E. Lhuillier, V. Brajuskovic, P. Guyot-Sionnest, *Nat. Photonics* **2011**, *5*, 489.
